# The Clinical Effectiveness of Single-Dose Human Papillomavirus Vaccination

**DOI:** 10.3390/vaccines12090956

**Published:** 2024-08-23

**Authors:** Wanying Bao, Xinlin He, Yue Huang, Rongyu Liu, Zhengyu Li

**Affiliations:** 1 Department of Gynecology and Obstetrics, West China Second University Hospital, Sichuan University, Chengdu 610041, China; baowanying614@stu.scu.edu.cn (W.B.); hxlapril22@163.com (X.H.); ambiguus@yeah.net (Y.H.); m13840700333@163.com (R.L.); 2 Key Laboratory of Birth Defects and Related Diseases of Women and Children, Department of Obstetrics and Gynecology, Ministry of Education, West China Second University Hospital, Sichuan University, Chengdu 610041, China

**Keywords:** human papillomavirus, vaccine, dose, effectiveness

## Abstract

The human papillomavirus (HPV) vaccine was initially approved for a three-dose regimen. Due to resource limitations, budget constraints, low acceptance, and poor adherence, global vaccination coverage is only 15%. A single-dose regimen could simplify logistics, reduce costs, and improve accessibility. However, its clinical effectiveness remains debatable. This review systematically searched PubMed, Embase, and Cochrane Library, including 42 clinical studies, to assess the effectiveness of a single-dose HPV vaccination for preventing HPV infections, cervical abnormalities, and genital warts. We summarized the effectiveness of bivalent, quadrivalent, and nonavalent vaccines across different age groups and buffer periods, and analyzed the factors contributing to the inconsistency of results. The review also provides insights into designing robust future research to inform single-dose HPV vaccination policies and guidelines, highlighting the need for further research to refine vaccination strategies.

## 1. Introduction

Human papillomavirus (HPV) infection is the most prevalent sexually transmitted infection globally, with an estimated 80% lifetime exposure risk [1]. Over 40 types of HPV can infect the genital regions, oral cavity, and throat in both men and women. Fifteen genotypes of HPV are linked to cervical cancer, and at least one type is associated with cancers of the vulva, vagina, penis, anus, and certain head and neck regions—specifically the oropharynx [2,3]. Recombinant prophylactic vaccines containing virus-like particles of HPV have been approved for human use, providing prevention against HPV infections, cervical abnormalities, genital warts, and anal intraepithelial neoplasia [2,4]. Despite these benefits, global vaccination coverage remains low at approximately 15% due to limited resources, budget constraints, low acceptance, and poor adherence to the HPV vaccine [5]. A single-dose regimen could simplify vaccine administration, lower costs, and increase accessibility compared to multi-dose schedules, and especially benefits low- and middle-income countries (LMICs), where 85% of cervical cancer deaths occur [6,7]. Thus, reliable and quantitative evidence on the effectiveness of a single-dose HPV vaccination is essential to guide decision-making.

While earlier studies have examined the efficacy of a single-dose HPV vaccination compared to two- and three-dose ones, the single-dose HPV vaccination schedule is still debatable [8,9,10]. Previous reviews on the clinical efficacy of the single-dose HPV vaccination have been limited by the inclusion of an incomplete range of studies, leading to insufficient reporting of clinical outcomes. In addition, these reviews did not explore the effectiveness of bivalent, quadrivalent, and nonavalent vaccines separately [8,9,11]. This called for a thorough analysis to evaluate and compare the clinical efficacy of single-dose regimen for each HPV vaccine variant. Our review included all eligible clinical studies, regardless of study design, aiming to assess the clinical effectiveness of a one-dose bivalent HPV vaccination (Cervarix^®^, Cecolin^®^ or Walrinvax^®^), quadrivalent HPV vaccination (Gardasil^®^), or nonavalent HPV vaccination (Cervarix^®^) [12] on HPV infection, cervical cytological and histological abnormalities, and genital warts in both men and women.

## 2. Methods

### 2.1. Search Strategy and Study Selection

To ensure a comprehensive search of the existing literature on the clinical effectiveness of the single-dose HPV vaccination, we searched PubMed, Embase, and Cochrane Library to identify eligible studies published before May 2024. Our search terms comprised (“papillomavirus vaccine”, or “papillomavirus vaccination”, “HPV vaccine”, “HPV vaccination”) AND (“vaccine effectiveness”, “infection”, “HPV”, “cervical abnormality”, “cervical neoplasm”, “cervical neoplasia”, “cervical intraepithelial neoplasia”, “HPV related disease”, “condyloma”, “genital wart”, “anogenital wart”, “anal intraepithelial neoplasia”) AND (“single-dose”, “one-dose”, “two-dose”, “three-dose”, “dose”), and detailed search strategies are given in Table S1. Moreover, we cross-referenced the references in the included studies to guarantee thorough coverage in the online search.

Studies were eligible for inclusion if they fulfilled the following criteria: (1) The study reported the clinical effectiveness of the HPV vaccination against HPV infection, cervical abnormalities, genital warts, and anal intraepithelial neoplasia. (2) The study compared the clinical effectiveness between the group receiving a one-dose HPV vaccine and the group not receiving an HPV vaccine, or between the group receiving a one-dose HPV vaccine and the group receiving a multiple-dose HPV vaccine. Studies were excluded if: (1) The study did not investigate the role of the prophylactic HPV vaccination. (2) The study solely explored the cost-effectiveness of vaccination. (3) The article type was a conference abstract or letter without original research data, or a review. (4) No full text could be found.

### 2.2. Data Extraction

Two authors (W.B. and X.H.) independently extracted the main study characteristics using standardized forms. Discrepancies were resolved by a third author (Z.L.). The main study characteristics included endpoint, vaccine, author, year, country, study design, gender, number of the population in each group, participant age, follow-up duration, case definition, assignment of dose number, buffer periods, sensitivity analyses by age group/buffer, adjustment, and outcome.

### 2.3. Data Synthesis

Data reported in the publications were synthesized narratively. The primary outcome was the effectiveness of HPV vaccination by comparing the HPV-related endpoints between individuals vaccinated with different numbers of doses (one vs. none, two vs. none, three vs. none). The vaccines analyzed included a bivalent HPV vaccine (Cervarix^®^, Cecolin^®^, or Walrinvax^®^), a quadrivalent HPV vaccine (Gardasil^®^), and a nonavalent HPV vaccine (Gardasil ^®^). Results were presented as crude or adjusted risk ratios (RR), hazard ratios (HR), incidence rate ratios (IRR), prevalence ratios (PR), or odds ratios (OR).

We decided not to use meta-analysis due to significant heterogeneity among the eligible studies: (1) The research encompassed both database-based investigations and clinical trials. The differences in their study topics, data sources, and methodological choices were substantial. (2) The study designs varied, including cohort studies, cross-sectional studies, and case-control studies. (3) The control groups exhibited inconsistency, as numerous studies compared non-vaccinated groups with 1-, 2-, and 3-dose vaccine groups, while other studies only compared groups receiving varying dosages. (4) Participants’ characteristics differed significantly, such as age at vaccination, sexual history, HPV infection status, and smoking status. (5) Clinical outcomes were measured at different follow-up times, leading to inconsistent evaluation time points for vaccine efficacy. (6) The results were reported in various formats, with some studies providing detailed numbers of patients, and others reporting only RR/HR/IRR/PR/OR. These distinctions between studies affected the consistency and comparability of the results, preventing standardization across studies. Therefore, we summarized the key information from each study in Table 1 and Tables S2–S4, and used forest plots to integrate useful data.

## 3. Results

### 3.1. Study Characteristics

**Figure 1:**
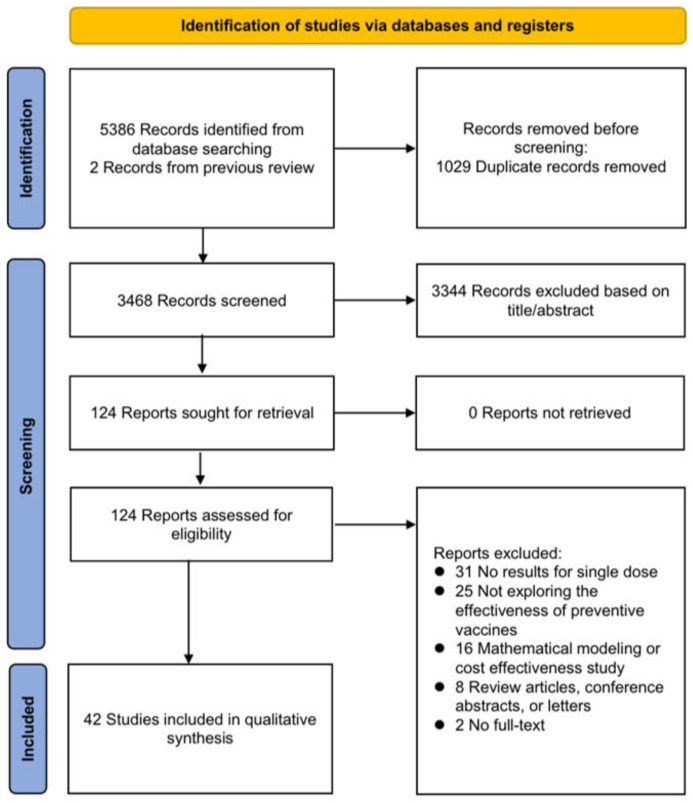
Flow diagram of study selection process.

A total of 5388 articles were initially identified. After a thorough screening process, 124 articles underwent full-text review, and 42 clinical studies published between 2011 and 2023 were included in the final analysis (Figure 1) [11,13,14,15,16,17,18,19,20,21,22,23,24,25,26,27,28,29,30,31,32,33,34,35,36,37,38,39,40,41,42,43,44,45,46,47,48,49,50,51,52,53]. Among these, 14 studies investigated the clinical effectiveness of the bivalent HPV vaccine, 29 studies focused on the quadrivalent HPV vaccine, and only two studies examined the efficacy of the nonavalent HPV vaccine. Regarding study populations, only one study on the quadrivalent vaccine included both men and women, and another study on the quadrivalent vaccine included males only, while the remaining studies included females only. The clinical outcomes assessed included cervical HPV infections, oral HPV infections, cervical cytological and histological abnormalities, and genital warts.

**Table 1:** Characteristics of studies that evaluated HPV vaccine effectiveness by number of doses.

Endpoint/ Vaccine/Author	Country	Study Design	Gender	Number of Population in Each Group	Participant Age		Follow-Up Duration	Case Definition	Assignment of Dose Number	Buffer Periods (Months)	Sensitivity Analyses by Age Group /Buffer
					Vaccination	Outcome					
Cervical HPV infection											
Bivalent vaccine											
Kreimer 2011 [21]	Costa Rica	Post-hoc analysis of RCT (CVT)	Women	0: 3578 1: 196 2 (months 0, 1 /0, 6): 422 3 (months 0, 1, 6): 2957	18–25	18–29	4 years	Persistent infection for 6/12 months: HPV 16/18	Final status	6	——^a^
Kavanagh 2014 [17]	Scotland	Cross-sectional study using screening registry data	Women	0: 3418 1: 55 2: 106 3: 1100	12–13	20–21	——	(1) HPV 16/18 (2) HPV 31/33/45 (3) HPV35/39/51/52/56/58/59 (4) Any HPV type	Final status	0	——
Kreimer 2015 [21]	Costa Rica	Retrospective cohort study (Combined CVT and PATRICIA data)	Women	1: case: 292 control: 251 2 (months 0, 1/0, 6): case: 611 control: 574 3 (months 0, 1, 6): case: 11,110 control: 11,217	15–25	15–29	4 years	Incident and persistent infection for 6/12 months: (1) HPV 16/18 (2) HPV 31/33/45	Final status	6	——
Cuschieri 2016 [22]	Scotland	Cross-sectional study using screening registry data	women	0: 3619 1: 177 2: 300 3: 1853	15–17	20–21	——	(1) HPV 16/18 (2) HPV 31/33/45	Final status	0	——
Kavanagh 2017 [27]	Scotland	Cross-sectional study using screening registry data	women	0: 4008 1: 223 2: 391 3: 3962	12–≥18	20–21	——	(1) HPV 16/18 (2) HPV 31/33/45 (3) HPV35/39/51/52/56/58/59 (4) Any HPV type	Final status	0	——
Safaeian 2018 [32]	Costa Rica	Post-hoc analysis of RCT (CVT)	Women	0: 2382 1: 134 2 (months 0, 1): 193 2 (months 0, 6): 79 3 (months 0, 1, 6): 2043	18–25	18–32	7 years	Year 7 prevalent HPV infection (an infection detected at year 7): (1) HPV 16/18 (2) HPV 31/33/45 (3) HPV 35/39/51/52/56/58/59 (4) Non-carcinogenic HPV	Final status	12	——
Kreimer 2020 [43]	Costa Rica	Post-hoc analysis of RCT (CVT)	Women	0: 1783 1: 112 2 (months 0, 6): 62 3 (months 0, 1, 6): 1365	18–25	18–37	12 years	Incident HPV infection (an infection detected at year 11 but not detected at the year or prevalent HPV infection (an infection detected at years 9 and 11: (1) HPV 16/18 (2) HPV 35/39/51/52/56/58/59 (3) Non-carcinogenic HPV	Final status	0	——
Barnabas 2022 [50]	Kenya	Randomized controlled trial	Women	0: 473 1: 489	15–20	15–22	18 months	Persistent HPV for ≥4 months: HPV 16/18	Final status	3, 6	Buffer
Barnabas 2023 [51]	Kenya	Randomized controlled trial	Women	0: 473 1: 489	15–20	15–23	3 years	Persistent HPV for ≥4 months: HPV 16/18	Final status	3, 6	Buffer
Quadrivalent vaccine											
Sankaranarayanan 2016 [25]	India	Prospective cohort study	Women	1: 870 2 (days 1, 60): 717 2 (days 1, ≥180): 526 3 (days 1, 60, ≥180): 536	10–18	10–22	4 years	Incident and persistent infection for ≥12 months: (1) HPV 16 (2) HPV 18 (3) HPV 16/18 (4) HPV 6/11 (5) HPV 6/11/16/18 (6) HPV 31/33/35 (7) HPV 6/11/16/18/31/33/35	Final status	0	——
Sankaranarayanan 2018 [33]	India	Prospective cohort study	Women	0: 1141 1: 604 2 (days 1, 60): 608 2 (days 1, ≥180): 818 3 (days 1, 60, ≥180): 959	10–18	10–25	7 years	Persistent infection for ≥12 months: (1) HPV 16/18 (2) HPV 31/33/35 (3) Non-vaccine targeted HPV	Final status	0	——
Sonawane 2019 [39]	United States	Cross-sectional study of a nationally representative sample	Women	0: 1004 1: 106 2: 126 3: 384	≤26	18–26	——	(1) HPV 6/11/16/18 (2) HPV 31/33/45 (3) HPV 35/39/51/55/56/58/59/68	Final status	0	——
Markowitz 2020 [44]	United States	Cross-sectional study of women enrolled in an integrated health-care delivery system	Women	0: 1052 1: 303 2: 304 3: 2610	≤29	20–29	——	(1) HPV 6/11/16/18 (2) Non-4vHPV (3) HPV 16/18/31/33/35/39/45/51 /52/56/58/59/66/68 (4) HPV 31/33/45	Final status	1	Age group
Batmunkh 2020 [41]	Mongolia	Retrospective cohort study	Women	0: 357 1: 118	11–17	16–26	6 years	(1) HPV 16 (2) HPV 18 (3) HPV 16/18 (4) Any HPV (5) HPV 35/39/51/52/56/58/59/68 (6) HPV 16 + other type/18 + other type/16 + 18 + other type	Final status	0	——
Basu 2021 [49]	India	Prospective cohort study	Women	0: 1479 1: 2858 2: 2166 3: 2019	10–18	10–28	10 years	Incident and persistent infections for ≥10 months: (1) HPV 16/18 (2) HPV 6/11/16/18 (3) HPV 31/33/35 (4) Any HPV	Final status	1	——
Reyburn 2023 [53]	Fiji	Retrospective cohort study	Women	0: 376 1: 158 2: 99 3: 189	15–23	15–34	6–11 years	(1) HPV 16/18/31/33/35/39/45 /51/52/56/58/59/66/68; (2) HPV 16/18 (3) HPV 6/11 (4) HPV 31/33/35/39/45/51/52 /56/58/59/66/68 (5) HPV 6/11/16/18/31/33/45/52/58 (6) HPV 6/11/31/33/45/52/58	Final status	0	——
Nonavalent vaccine											
Barnabas 2022 [50]	Kenya	Randomized controlled trial	Women	0: 473 1: 496	15–20	15–22	18 months	Persistent HPV infection for ≥4 months: (1) HPV 16/18 (2) HPV 16/18/31/33/45/52/58	Final status	3, 6	Buffer
Barnabas 2023 [51]	Kenya	Randomized controlled trial	Women	0: 473 1: 496	15–20	15–23	3 years	Persistent HPV infection for ≥4 months: (1) HPV 16/18 (2) HPV 16/18/31/33/45/52/58 (3) HPV 31/33/45	Final status	3, 6	Buffer
Penile, scrotal, or anal HPV infection in men											
Quadrivalent vaccine											
Widdice 2019 [40]	United States	Cross-sectional study	Men	0: 471 1: 58 2: 37 3: 143	Attained vaccine in 2013–2014: 16.2 (mean); attained vaccine in 2016–2017: 15.1 (mean)	13–26	——	HPV 6/11/16/18	Final status	0	——
Oral HPV infection in women											
Quadrivalent vaccine											
Gheit 2023 [52]	India	Retrospective cohort study	Women	0: 179 1: 204 2 (days 1 and 60): 101 3 (days 1 and 180): 190 3:(days 1, 60, and 180): 323	10–18	18–25	——	(1) HPV 16 (2) HPV 18 (3) HPV 16/18 (4) HPV 6/11/16/18 (5) Non-HPV 6/11/16/18 (6) Any HPV	Final status	0	——
Cervical abnormalities											
Bivalent vaccine											
Pollock 2014 [18]	Scotland	Retrospective cohort study using linked national registry data	Women	0: 76,114 1: 1315 2: 2725 3: 25,898	15–>18	20–21	——	Histology: (1) CIN3 (2) CIN2 (3) CIN1	Final status	0	——
Cameron 2017 [26]	Scotland	Retrospective cohort study using linked national registry data	Women	0: 75,683 1: 2258 2: 4462 3: 55,303	14–>18	20–21	——	Histology: (1) CIN3 (2) CIN2 (3) CIN1	Final status	0	——
Palmer 2019 [38]	Scotland	Retrospective cohort study using linked national registry data	Women	0: 64,026 1: 2051 2: 4135 3: 68,480	12–>18	20	——	Histology: (1) CIN3+ (2) CIN2 (3) CIN1 Cytology: (1) high grade dyskaryosis/HSIL-H borderline/ASCUS (2) moderate dyskaryosis/HSIL-M (3) low grade dyskaryosis/LSIL (4) borderline/ASCUS	Final status	0	——
Martellucci 2021 [47]	Italy	Retrospective cohort study using administrative data	Women	0: 7394 1: 212 2: 83 3: 96	14–>30	17–32	——	Cytology: (1) HSIL (2) LSIL	Final status	1, 6, 12	Buffer
Quadrivalent vaccine											
Gertig 2013 [14]	Australia	Retrospective cohort study using linked data from registries	Women	0: 14,085 1: 1422 2: 2268 3: 21,151	12–19	12–21	——	Histology: (1) CIN3/AIS (2) CIN2+/AIS (3) CIN2 (4) CIN1 Cytology: (1) High grade (possible HSIL, HSIL, HSIL with possible microinvasion/invasion, squamous cell carcinoma, possible high-grade endocervical glandular lesion, AIS, AIS with possible microinvasion/invasion and adenocarcinoma) (2) Low grade (possible LSIL, LSIL and atypical endocervical cells of uncertain significance)	Time-dependent; final status	0	——
Crowe 2014 [15]	Australia	Case-control study using linked data from registries	Women	0: 60,282 1: 10,879 2: 12,073 3: 25,119	12–26	11–31	——	Histology: (1) CIN2+/AIS (2) Other cases (either a low-grade abnormality at histology or an abnormal cytology result that was not confirmed by histology)	Final status	0, 1, 6, 12	Age group, buffer
Brotherton 2015 [11]	Australia	Retrospective cohort study using linked regional data registries	Women	0: 133,055 1: 20,659 2: 27,500 3: 108,264	12–26	12–30	——	Histology: (1) CIN3/AIS, (2) CIN2+ (3) CIN2 Cytology: (1)high grade (possible HSIL, HSIL, HSIL with possible microinvasion/invasion, squamous cell carcinoma, possible high-grade endocervical glandular lesion, AIS, AIS with possible microinvasion/invasion and adenocarcinoma) (2) low grade (possible LSIL, LSIL)	Final status	0, 1, 6, 12, 24	Age group, buffer
Hofstetter 2016 [23]	United States	Retrospective cohort study using medical center records	Women	0: 1632 1: 695 2: 604 3: 1196	11–20	11–27	——	Cytology: Any abnormal (atypical squamous cells of undetermined significance, atypical glandular cells, atypical squamous cells cannot exclude a high-grade squamous intraepithelial lesion, low-grade squamous intraepithelial lesion, and high-grade squamous intraepithelial lesion)	Final status	1	Age group
Kim 2016 [24]	Canada	Nested case-control study using linked data from registries	Women	0: 5712 1: 327 2: 490 ≥3: 3675	10–15	18–21	——	Cytology: (1) High grade (ASC-H or HSIL) (2) Low grade (ASC-US or LSIL) (3) Abnormal (ASC-US, LSIL, ASC-H or HSIL)	Final status	0	——
Silverberg 2018 [34]	United States	Nested case-control study of women enrolled in an integrated health-care delivery system	Women	0: 23,293 1: 756 2: 554 ≥3: 1527	14–26	18–34	——	Histology: (1) CIN3+ (2) CIN2+	Final status	6	——
Dehlendorff 2018 [30]	Denmark & Sweden	Retrospective cohort study using linked national registry data	Women	0: 2,091,579 1: NA 2: NA 3: NA	13–29	13–30	——	Histology: CIN2+	Time- dependent	0	Age group
Brotherton 2019 [37]	Australia	Retrospective cohort study using linked regional data registries	Women	0: 48,845 1: 8618 2: 18,190 3: 174,995	≤13–22	15–22	——	Histology: (1) CIN3/AIS + histopathology (2) CIN2+/AIS	Final status	0, 12, 24	Buffer
Verdoodt 2020 [46]	Denmark	Retrospective cohort study using linked national registry data	Women	0: 374,327 1: 10,480 2: 30,259 3: 174,532	12–16	17–25	——	Histology: (1) CIN3+ (2) CIN2+	Time- dependent	0, 6 in secondary analysis	Age group
Johns 2020 [42]	United States	Case-control study using medical records data	Women	0: 2731 1: 136 2: 108 3: 325	12–26	18–39	——	Histology: CIN2+	Final status	1, 12, 24, 36	Buffer
Rodriguez 2020 [45]	United States	Retrospective matched cohort study using health insurance claims database	Women	0: 66,541 1: 13,630 2: 14,088 ≥3: 38,823	9–26	9–31	——	Histology: CIN2/3 Cytology: high-grade (HSIL/ASC-H)	Final status	12	Age group, dose interval, history and results of pap test
Genital warts											
Bivalent vaccine											
Navarro- Illana 2017 [28]	Spain	Retrospective cohort study using national health registries	Women	0: 607,006 1: 18,142 2: 31,420 3: 153,296	14	14–19	——	Genital warts (ICD9-CM code 078.11)	Time- dependent	0	——
Quadrivalent vaccine											
Herweijer 2014 [16]	Sweden	Retrospective cohort study using population-based health registries	Women	0: 1,045,157 1: 115,197 2: 107,338 3: 89,836	10–19	10–24	——	Genital warts (ICD-10 code A63.0 or podophyllotoxin/imiquimod prescription)	Time- dependent	0–12	Age group
Blomberg 2015 [19]	Denmark	Retrospective cohort study using national health registries	Women	0: 188,956 1: 55,666 2: 93,519 3: 212,549	12–27	12–27	——	Genital warts (ICD-10 code A63.0 or podophyllotoxin prescription)	Time- dependent	1	——
Dominiak- Felden 2015 [30]	Belgium	Retrospective cohort study using sick-fund/insurance reimbursement database	Women	0: 63,180 1: 4020 2: 3587 3: 35,792	10–22	16–22	——	Genital warts (reimbursement of imiquimod)	Time- dependent	1	——
Perkins 2017 [29]	United States	Retrospective cohort study using commercial claims database	Women	0: 201,933 1: 30,438 2: 36,583 3: 118,962	9–25	9–25	——	Genital warts (ICD-9 and CPT codes and prescriptions)	Final status	0	——
Navarro- Illana 2017 [28]	Spain	Retrospective cohort study using national health registries	Women	0: 607,006 1: 18,142 2: 31,420 3: 153,296	14	14–19	——	Genital warts (ICD-9-CM code 078.11)	Time- dependent	0	——
Hariri 2018 [31]	United States	Retrospective cohort study in integrated health-care delivery systems	Women	0: 31,563 1: 5864 2: 5459 3: 21,631	16–17 (mean)	11–28	——	Genital warts (ICD-9 code 078.10, 078.11, 078.19, specialty of diagnosing provider, and sexually transmitted infection tests ordered)	Final status	6 from last dose 12 from first dose	Buffer
Zeybek 2018 [36]	United States	Matched retrospective cohort study using health insurance claims databases	Men and women	0: 286,963 1: 54,280 2: 55,632 3: 177,051	9–26	10–31	5 years	Genital warts (ICD-9-CM or 10 code 078.11 or A63.0)	Final status	3 from the last dose	Age group
Willows 2018 [54]	Canada	Matched retrospective cohort study using linked vaccine registry and claims and population-based databases	Women	0: 94,327 1: 3521 2: 6666 3: 21,277	9–26	10–33	——	Genital warts (ICD-9-CM or 10 code 078.11 or A63.0 and related procedure code)	Final status	0	Age group
Baandrup 2021 [48]	Denmark	Retrospective cohort study using population-based health national registries	Women	0: 1,904,895 1: 235,653 2: 460,978 3: 1,934,589 (person-yrs)	12–30	12–30	——	Genital warts (ICD-10 code A63.0 or podophyllotoxin prescription)	Time- dependent	1	Age group, maternal educational level
Reyburn 2023 [53]	Fiji	Retrospective cohort study	Women	0: 376 1: 158 2: 99 3: 189	15–23	15–34	6–11 years	Genital warts (confirmed by the midwife and antenatal clinic obstetric doctor)	Final status	0	——

Abbreviations: HPV, human papillomavirus; CIN, cervical intraepithelial neoplasia; AIS, adenocarcinoma in situ; HSIL, high-grade squamous intraepithelial lesions (HSIL-M = mild dyskaryosis; HSIL-H = high grade dyskaryosis); LSIL, low-grade squamous intraepithelial lesions; ASC-H, atypical squamous cells, cannot exclude HSIL; ASC-US, atypical squamous cells of undetermined significance; ICD-9-CM, international classification of diseases, 9th revision, clinical modification; CPT, current procedural terminology. ^a^ Articles did not have sensitivity analyses by age group/buffer.

### 3.2. Cervical HPV Infection in Women

#### 3.2.1. Bivalent Vaccine

The HPV 16/18 infection in the one-dose group was evaluated in nine studies. According to three trials from a single piece of research, the one-dose bivalent vaccine offered comparable protection against HPV 16/18 infection to the two- and three-dose regimens across follow-up periods of 4, 7, and 12 years [21,32,43]. Besides, the effectiveness of the single-dose vaccination in preventing persistent HPV 16/18 infections for at least 4 months [50,51], 6 months [13,21], and 12 months [13,21] was similar to that of two and three doses. Nevertheless, three studies reported that a single dose was less beneficial than two and three doses [17,22,27]. Notably, Barbanas et al.’s studies evaluated the vaccine’s effectiveness by applying varying buffer periods and suggested that the effectiveness of a single dose increased with a 6-month buffer period compared to 3 months [50,51].

Six studies evaluated the single-dose bivalent vaccination’s effectiveness against HPV 31/33/45 infections, but only that of Safaeian et al. showed similar protection for one, two, and three doses [32]. Four studies assessed the vaccine’s impact on high-risk HPV (hrHPV) 35/39/51/52/56/58/59, finding no significant prevention effectiveness, regardless of the number of doses [17,27,32,43].

#### 3.2.2. Quadrivalent Vaccine

Four studies concluded that a single dose was highly effective against HPV16/18 infection [25,41,49,53]. Three studies comparing the effectiveness of 1, 2, and 3 doses against 10 months and ≥12 months of persistent HPV16/18 infection also found similar protection across all doses [25,33,49].

Four studies evaluated the vaccine’s efficacy against HPV 6/11/16/18, indicating similar protection for 1, 2, and 3 doses [25,39,44,49]. One study noted higher effectiveness among women who received the first dose at age ≤ 18 than those >18, regardless of the number of doses [44].

The effectiveness of quadrivalent vaccine against HPV31/33/45 varied across five studies [25,33,39,44,49]. Only one study reported lower HPV31/33/45 infection rates in the one-dose group compared to unvaccinated individuals, similar to the two- and three-dose groups. Three studies indicated no protection against HPV31/33/45 with one dose [25,39,44], and two studies based on the same clinical study showed that one-dose quadrivalent vaccination could not protect against persistent HPV 31/33/45 infection over 12 months during the 4-year and 7-year follow-ups [25,33].

Two studies provided data on all hrHPV (16/18/31/33/35/39/45/51/52/56/58/59/66/68) [44,53], and one study provided data on non-vaccine hrHPV (31/33/35/39/45/51/52/56/58/59/66/68) [53]. They all showed that one-, two-, and three-dose quadrivalent vaccinations did not reduce the hrHPV infection rate.

#### 3.2.3. Nonavalent Vaccine

Two manuscripts based on the same study explored the effectiveness of a nonavalent vaccine on HPV infection, and both analyzed the vaccine effectiveness with different buffer periods [50,51].

The efficacy of the single-dose nonavalent HPV vaccination was high among persistent HPV 16/18 and persistent HPV 16/18/31/33/45/52/58 for at least 4 months in 18-month [50] and 3-year [51] follow-up periods, and high among persistent HPV31/33/45 for at least 4 months in a 3-year follow-up duration [51], exhibiting strong defenses against vaccine-specific HPV infection. Both of these publications showed that the efficacy of a single dose appeared to rise with buffer periods of six months as opposed to three months.

**Figure 2:**
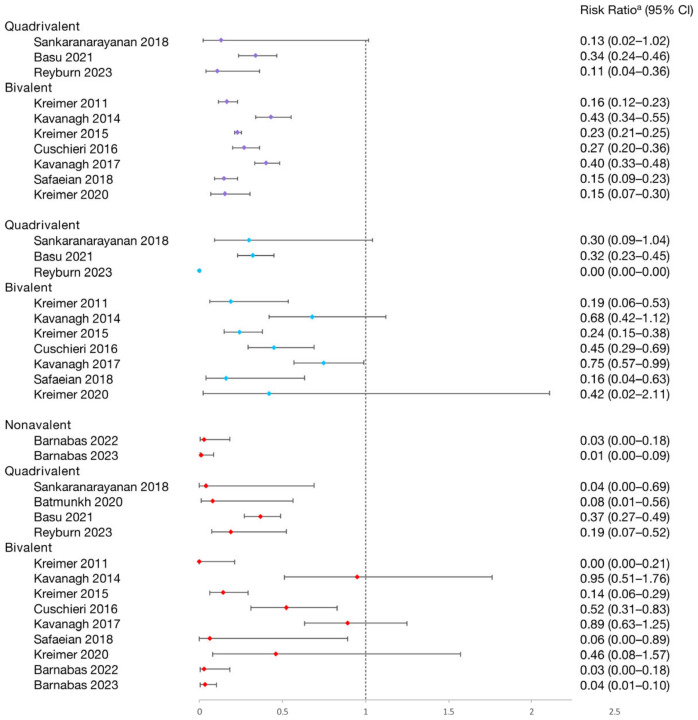
Effectiveness of varying doses of bivalent, quadrivalent, and nonavalent HPV vaccines in protecting women against HPV 16/18 infection [13,17,21,22,27,32,33,43,49,50,51,53]. Purple square: 3 dose vs. 0 dose; blue square: 2 doses vs. 0 dose; red square: 1 dose vs. 0 dose. ^a^ The risk ratio encompasses various measures depending on the study, including risk ratio, hazard ratio, prevalence ratio, or odds ratio. The risk ratio for Sankaranarayanan 2018 and Safaeian 2018 was not provided in the original texts and was calculated based on sample sizes. Other risk ratios in the figure were extracted directly from the original articles. If the original articles did not include available data for all age groups, they were excluded from this figure.

**Figure 3:**
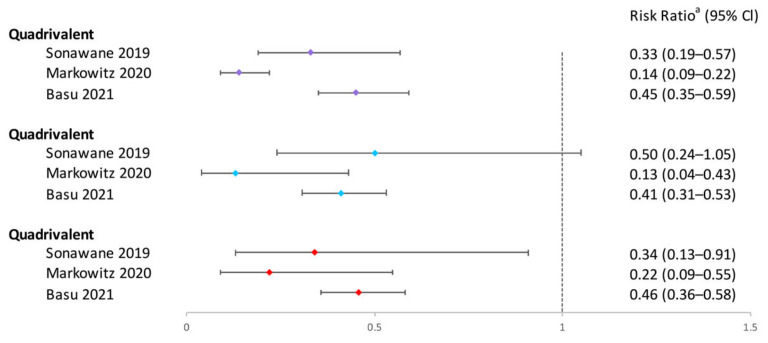
Effectiveness of varying doses of quadrivalent HPV vaccine in protecting women against HPV 6/11/16/18 infection. Purple square: 3 dose vs. 0 dose; blue square: 2 doses vs. 0 dose; red square: 1 dose vs. 0 dose [39,44,49]. ^a^ The risk ratio encompasses various measures depending on the study, including risk ratio, prevalence ratio, or hazard ratio. The risk ratio for Sonawane 2019 was not provided in the original texts and was calculated based on sample sizes. Other risk ratios in the figure were extracted directly from the original articles. If the original articles did not include available data for all age groups, they were excluded from this figure.

**Figure 4:**
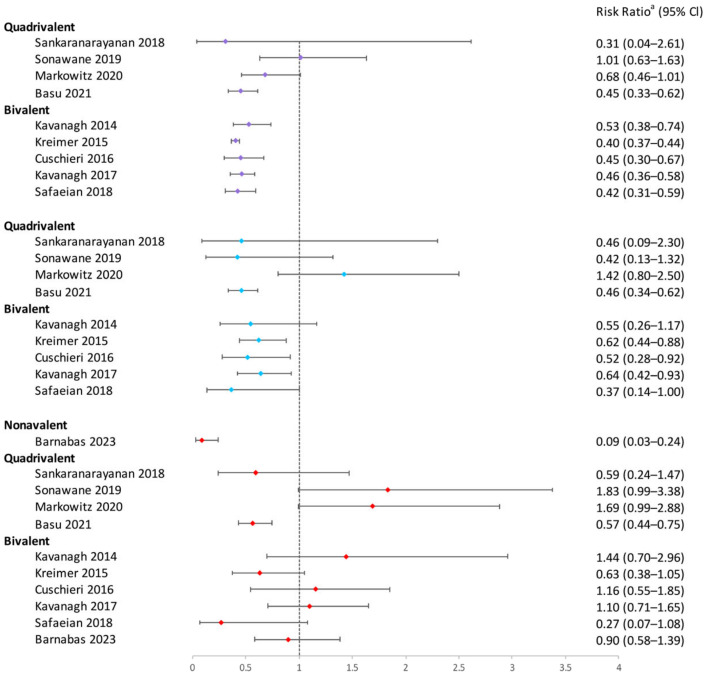
Effectiveness of varying doses of bivalent, quadrivalent, and nonavalent HPV vaccines in protecting women against HPV 31/33/35 infection. Purple square: 3 dose vs. 0 dose; blue square: 2 doses vs. 0 dose; red square: 1 dose vs. 0 dose [17,21,22,27,32,33,39,40,49,51]. ^a^ The risk ratio encompasses various measures depending on the study, including risk ratio, prevalence ratio, hazard ratio, or odds ratio. The risk ratio for Sankaranarayanan 2018, Sonawane 2019, and Safaeian 2018 was not provided in the original texts and was calculated based on sample sizes. Other risk ratios in the figure were extracted directly from the original articles. If the original articles did not include available data for all age groups, they were excluded from this figure.

In conclusion, the one-dose bivalent vaccination was able to prevent HPV16/18 infections, although some research suggested that two or three doses of bivalent vaccination were more effective (Figure 2). One-dose quadrivalent and nonavalent HPV vaccinations showed comparable protection against vaccine-specific HPV types to two- and three-dose regimens, with the effectiveness rising with younger age groups and longer buffer periods (Figure 2 and Figure 3). Comparing single-dose bivalent or quadrivalent HPV vaccinations to two and three doses, single-dose vaccination demonstrated less cross-protection against hrHPV (Figure 4).

### 3.3. Other HPV Infection

#### 3.3.1. Penile, Scrotal, or Anal HPV Infection in Men

Among young men who had previously engaged in sexual activity, a cross-sectional study examined the efficacy of the quadrivalent vaccine [40]. The results showed no statistically significant differences in vaccine-type HPV infection rates between those receiving 0, 1, 2, or ≥3 doses.

#### 3.3.2. Oral HPV Infection in Women

A retrospective cohort study aimed to investigate whether married female participants could avoid oral HPV infection by taking the quadrivalent vaccine [52]. The findings implied that vaccine-type and non-vaccine-type oral HPV infection might be avoided with two- or three-dose HPV vaccination, but not with a one-dose one.

### 3.4. Cervical Histological and Cytological Abnormalities in Women

#### 3.4.1. Bivalent Vaccine

Four studies assessed the effectiveness of the bivalent vaccine in reducing cervical histological abnormalities. Two studies showed no significant effectiveness for one or two doses on reducing atypical squamous cells of undetermined significance (ASC-US), low-grade squamous intraepithelial lesions (LSIL), high-grade squamous intraepithelial lesions (HSIL), cervical intraepithelial neoplasia 1 (CIN1), CIN2+, and CIN3+, with only three doses being effective except for LSIL [18,38]. Cameron et al. [26] found that the one-dose quadrivalent vaccine could reduce CIN1, CIN2, and CIN 3, but the effect was weaker than with two or three doses. Only Martellucci et al. found reductions in LSIL for at least one dose, similar to two and three doses, with effectiveness confirmed across buffer periods [47].

#### 3.4.2. Quadrivalent Vaccine

Data on the prevention of cervical histological and cytological abnormalities with a single dose of quadrivalent vaccine have been reported in 11 studies [11,14,15,23,24,30,34,37,42,45,46]. The effectiveness of a quadrivalent vaccine against CIN3+,CIN2+, high-grade (HG) abnormalities, and cytological low-grade (LG) abnormalities all varied across different studies. Some found that one-, two-, and three-dose vaccinations provided similar protection [45,46], and some noted that single-dose effectiveness was slightly weaker than two- and three-dose regimens [15,24,30,37,42], while other studies reported that a single dose was not effective [34].

In studies that support the effectiveness of single-dose immunization, five studies evaluated the effectiveness by using varying age groups [11,15,30,45,46]. Two studies reported that the quadrivalent vaccine had better effectiveness on younger age groups. Verdoodt et al. found a lower CIN3+ rate in women aged under 23, compared to those aged 23 and older [46]. Dehlendorff et al. reported that CIN2+ risk decreased in women aged 13–16 and 17–19, but that it could not decrease in women aged 20–29 [30]. Notably, three studies found the quadrivalent vaccine was less effective in not only older women, but also in young girls. Crowe et al. found the vaccine effective against CIN2+ among women vaccinated at ages 15–18 and 19–22, but not among those aged 11–13 and 23–27 [15]. Rodriguez et al. found a similar reduction in HG abnormalities for women aged 15–19, but no effectiveness in those under 15 or over 20 [45]. Brotherton et al. observed a decrease in HG abnormalities in women vaccinated at ages 17–23, but no decrease for women aged ≤ 16 [11].

In studies suggesting the efficacy of single-dose vaccination, the impact of different buffer intervals on vaccine efficacy was examined in four studies [11,15,37,42]. They all discovered that extended buffer intervals increased vaccination efficacy.

**Figure 5:**
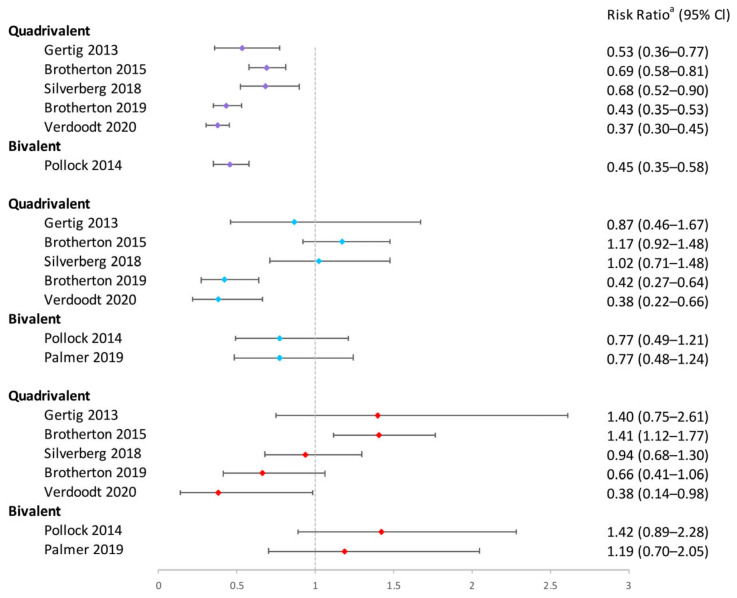
Effectiveness of varying doses of bivalent and quadrivalent HPV vaccines in protecting women against CIN3+ [11,14,18,34,37,38,46]. Purple square: 3 doses vs. 0 dose; blue square: 2 doses vs. 0 dose; red square: 1 dose vs. 0 dose. ^a^ The risk ratio encompasses various measures depending on the study, including hazard ratio, risk ratio, incidence rate ratio, or odds ratio. The risk ratio in the figure was extracted directly from the original articles. If the original articles did not include available data for all age groups, they were excluded from this figure.

**Figure 6:**
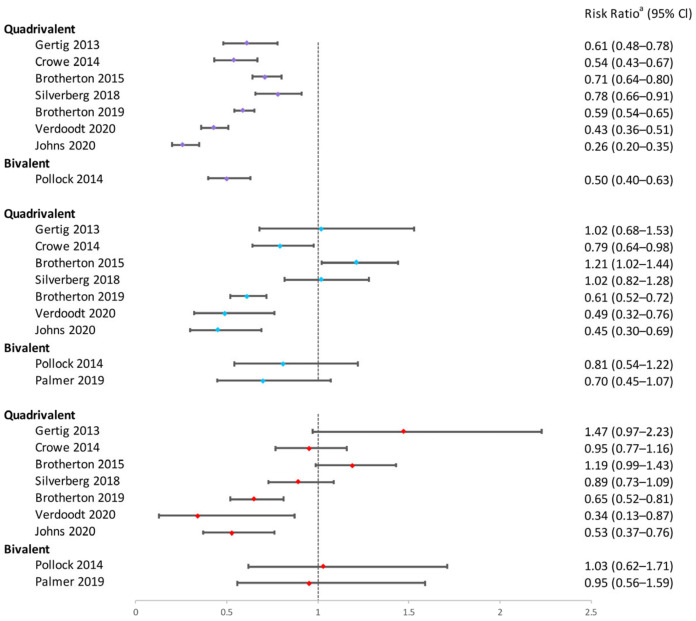
Effectiveness of varying doses of bivalent and quadrivalent HPV vaccines in protecting women against CIN2+. Purple square: 3 doses vs. 0 dose; blue square: 2 doses vs. 0 dose; red square: 1 dose vs. 0 dose [11,14,15,18,34,37,38,42,46]. ^a^ The risk ratio encompasses various measures depending on the study, including hazard ratio, odds ratio, risk ratio, or incidence rate ratio. The risk ratio in the figure was extracted directly from the original articles. If the original articles did not include available data for all age groups, they were excluded from this figure.

**Figure 7:**
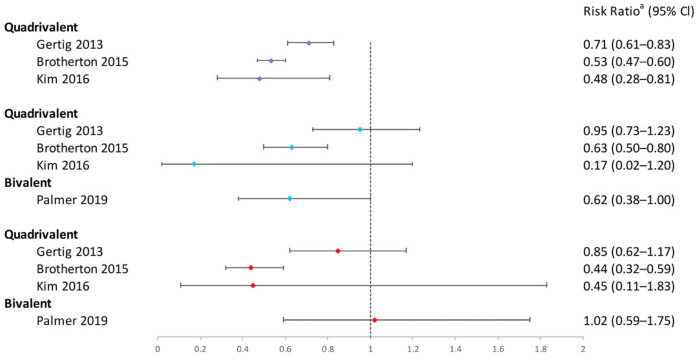
Effectiveness of varying doses of bivalent and quadrivalent HPV vaccines in protecting women against cytological high-grade abnormalities [11,14,24,38]. Purple square: 3 doses vs. 0 dose; blue square: 2 doses vs. 0 dose; red square: 1 dose vs. 0 dose. ^a^ The risk ratio encompasses various measures depending on the study, including hazard ratio or odds ratio. The risk ratio in the figure was extracted directly from the original articles. If the original articles did not include available data for all age groups, they were excluded from this figure.

**Figure 8:**
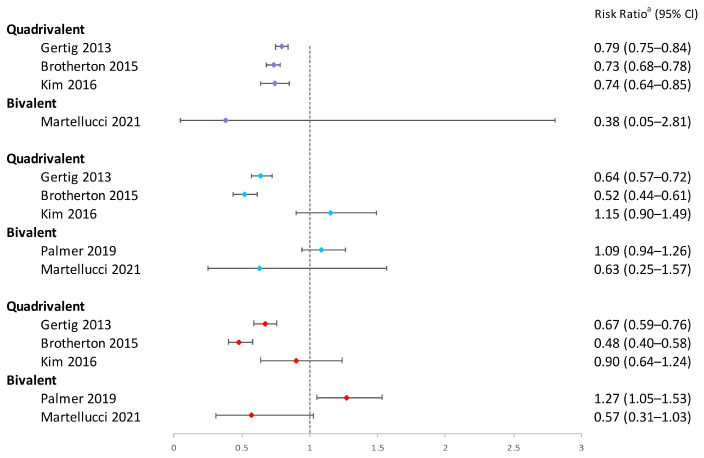
Effectiveness of varying doses of bivalent and quadrivalent HPV vaccines in protecting women against cytological low-grade abnormalities [11,14,24,38,47]. Purple square: 3 doses vs. 0 dose; blue square: 2 doses vs. 0 dose; red square: 1 dose vs. 0 dose. ^a^ The risk ratio encompasses various measures depending on the study, including hazard ratio or odds ratio. The risk ratio in the figure was extracted directly from the original articles. If the original articles did not include available data for all age groups, they were excluded from this figure.

To sum up, single-dose bivalent HPV vaccinations showed limited effectiveness in reducing cervical abnormalities compared to three-dose ones; single-dose quadrivalent HPV vaccines demonstrated varying efficacy in preventing cervical abnormalities, while two and three doses of vaccination generally provided more consistent protection (Figure 5, Figure 6, Figure 7 and Figure 8). Better effectiveness of bivalent and quadrivalent HPV vaccines could be found in studies with longer buffer periods.

### 3.5. Genital Warts in Women and Men

Ten studies examined the association between a one-dose quadrivalent HPV vaccination and the first occurrence of condyloma [16,19,20,28,29,31,35,36,48,53]. Eight studies indicated the greatest reduction in condyloma risk with three doses and the least with one dose [16,19,20,28,29,31,35,48]. Notably, the outcomes of a retrospective cohort study involving young pregnant women and a follow-up of a duration of 6–11 years contradicted the previously mentioned research findings [53]. However, the 95% confidence intervals for the aPR crossed the null value because of the limited case numbers.

The vaccine effectiveness was analyzed with various age groups in four studies [16,35,36,48]. Three studies demonstrated that younger age groups had lower genital wart rates [16,36,48,54]. It is noteworthy that Zeybek et al. found similar effectiveness for one, two, and three doses in preventing genital warts among 15–19-year-olds, but weaker effectiveness for those under 15, and no reduction in warts for women aged ≥ 20 [36].

The vaccine effectiveness was analyzed with different buffer periods only in Hariri et al.‘s study. According to their sensitivity analysis, longer buffer durations for all dose groups were associated with higher vaccine effectiveness [31].

**Figure 9:**
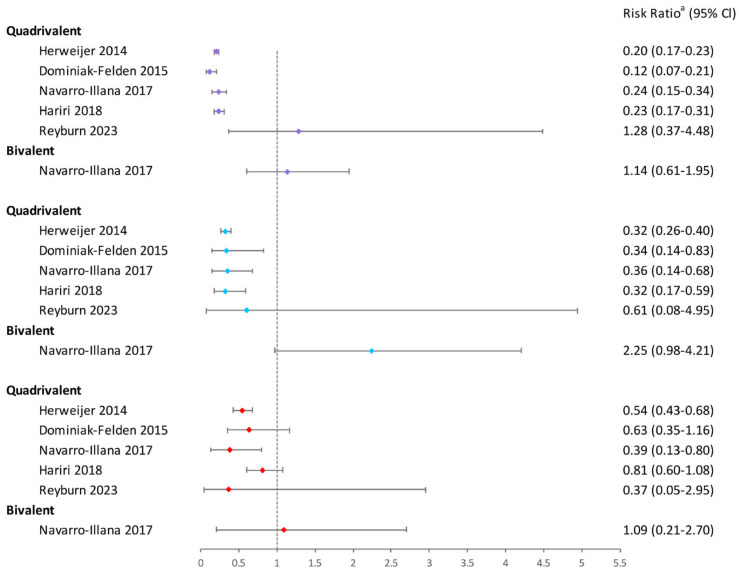
Effectiveness of varying doses of bivalent and quadrivalent HPV vaccines in preventing women against genital warts [16,20,28,31,53]. Purple square: 3 doses vs. 0 dose; blue square: 2 doses vs. 0 dose; red square: 1 dose vs. 0 dose. ^a^ The risk ratio encompasses various measures depending on the study, including incidence rate ratio, risk ratio, hazard ratio, or prevalence ratio. The risk ratio in the figure was extracted directly from the original articles. If the original articles did not include available data for all age groups, they were excluded from this figure.

To sum up, when compared to two or three doses, the single-dose quadrivalent HPV vaccination is less efficient at preventing genital warts (Figure 9). Longer buffer times and lower age could help increase the efficacy of the quadrivalent HPV vaccine. In addition, there was just one trial that examined the prevention of genital warts with a bivalent HPV vaccine, and the findings indicated that there was no protection against the disease, regardless of the dosage administered [28].

## 4. Discussion

Public health policies prioritize enhancing the effectiveness of vaccination, maximizing coverage in target populations, addressing vaccine supply shortages, and facilitating cost-effective vaccine delivery [54]. Administering a single dose of the HPV vaccine offers significant advantages over the traditional three-dose regimen, including potentially higher uptake, fewer side effects, better cost-effectiveness, and more simplified logistics. It is crucial to have robust and quantitative data on the efficacy of a single-dose HPV vaccination. Our review provided robust evidence suggesting that one, two, or three doses of quadrivalent and nonavalent HPV vaccinations offered similar protective effectiveness against vaccine-type HPV infection. One dose of a bivalent vaccination could prevent HPV16/18 infections, although some studies indicated it was less effective than two or three doses. There was ongoing debate regarding the prevention of cervical abnormalities with one-dose bivalent or quadrivalent vaccinations. Additionally, two or three doses of a quadrivalent vaccination were more beneficial than one dose for preventing genital warts. Notably, the efficacy of the HPV vaccine declined with increasing age and extending the buffer period.

HPV16 and HPV18 are the most prevalent types found in cervical cancer patients, accounting for approximately 71% of cervical cancer worldwide [55]. There was continuous discussion regarding the HPV16/18 infection rate among participants receiving one, two, or three doses of the HPV vaccine. A review of clinical trials specifically designed to randomize participants to receive a single dose of the HPV vaccine found no significant difference in HPV16/18 infection rates between the one-, two-, and three-dose groups [9]. However, a meta-analysis concluded that the risk of HPV16/18 infection was slightly higher in the one-dose group compared to those receiving multiple doses [11]. Based on a more thorough literature analysis, we found that a single dosage of quadrivalent and nonavalent vaccinations was just as efficient at preventing HPV16/18 infection as two and three doses. Further research is needed to evaluate the efficacy of a single dose of bivalent vaccination.

In addition to HPV16 and HPV18, quadrivalent and nonavalent vaccines target HPV6 and HPV11, which cause over 90% of genital warts [56]. Similar to two and three doses, single doses of both 4-valent and 9-valent vaccines could effectively prevent HPV 6 and 11 infections. Nonavalent vaccines also target hrHPV 31, 33, 45, 52, and 58, which are responsible for up to 90% of additional cervical cancer globally [55,57]. Our findings suggest that the efficacy of bivalent or quadrivalent vaccines in preventing hrHPV infections was limited, regardless of the dosage, while a single dose of nonavalent vaccination could offer similar protection against HPV16/18/31/33/45/52/58 as two and three doses. This evidence underscored the importance of the 9-valent HPV vaccine in mitigating hrHPV infection [58,59].

Invasive cervical cancer, preceded by a long phase of precancerous lesions, is a leading cause of cancer-related deaths among women in LMIC [60]. Without treatment, more than 20% of CIN2/3 lesions progress to carcinoma in situ or cancer [61]. A meta-analysis found that a one-dose HPV vaccination had a similar impact on preventing HSIL, ASC-H, or CIN2/3 as multiple doses, although study heterogeneity was high [11]. However, a review summarized that only some recently published studies had reported that effectiveness with three, two and one doses was similar [8]. Our review found variability in bivalent and quadrivalent vaccines’ roles in preventing CIN3+, CIN2+, HG, and LG lesions across studies. Furthermore, while a single dose of quadrivalent vaccination could prevent genital warts, its effectiveness was weaker than that of two or three doses. These results highlight the requirement for more doses.

Current evidence indicates that vaccinating younger individuals is more effective. This is because younger participants are more likely to be sexually inactive, reducing the likelihood of pre-existing HPV infections at the time of vaccination, and the immune response to the HPV vaccine is robust in HPV-naive cohorts [62]. In several studies [30,45,48], women vaccinated at age ≥ 19 or 20 showed no significant difference in the risk of histologically confirmed HG lesions compared to unvaccinated women, likely due to pre-vaccination exposure to hrHPV. However, in some studies [11,15,36,45], for girls vaccinated before age 16, there was no significant difference in the risk of cytologically or histologically cervical abnormalities compared to unvaccinated girls, possibly due to the lack of ability to study this outcome in such a young group where events were rare.

Our research and previous studies indicated that the effectiveness of HPV vaccination increased with longer buffer periods. The possible reason is that partially vaccinated women were often older than fully vaccinated women, making them more likely to have pre-vaccination HPV infections [24,45,46]. Many studies lacked baseline information on HPV DNA status, HPV serology, or sensitive indicators of previous HPV exposure [14,15,20,24,28,29,30,31,35,36,46,48], potentially causing the effectiveness of one- and two-dose vaccinations to be underestimated. Buffer periods, which delay case counting, help address this issue by increasing the likelihood that detected outcomes are due to post-vaccination infections.

Our study is distinguished from prior reviews by incorporating all eligible studies on single-dose HPV vaccinations, exploring the effectiveness of bivalent, quadrivalent, and nonavalent vaccines, and analyzing various clinical outcomes, including cervical and oral vaccine-type HPV infections, non-vaccine-type hrHPV infections, both cytological and histological cervical abnormalities, as well as genital warts.

Several factors might contribute to any remaining inconsistency in our analysis: (1) Some studies that used information from commercial claim databases and population-based health registries might have incorrectly classified people’s vaccination statuses and baseline characteristics if they received care for a disease outside of their insurance or health plan, or if they did not take part in disease screening and treatment. (2) Many studies did not have accurate data regarding sexual behavior and baseline HPV infection status. (3) Regarding unmeasured or missing characteristics, women may have varied among research and groups (i.e., with respect to risk behaviors or smoking status). (4) The variability in the design, implementation, and reporting quality of the included studies may introduce study quality bias, leading to systematic errors. (5) Some non-English articles may not be published in the searched databases, leading to language bias in the review.

However, our analysis has inherent limitations: (1) Due to the limited number of studies on nonavalent vaccines, we could not fully analyze the clinical efficacy of a single dose of the 9-valent vaccine. (2) Many studies lacked data on comorbidities in the study populations prior to vaccination, preventing an analysis of the vaccine effectiveness on specific populations at higher risk of HPV infection and precancerous lesions, such as HIV patients, cancer patients, and organ transplant recipients [63]. (3) Few studies investigated the protective effectiveness of the HPV vaccine against oral HPV infections, male genital HPV infections, and male genital warts, limiting our ability to thoroughly explore the vaccine effectiveness.

Consequently, more pragmatic studies with rigorous designs are warranted to ascertain the single-dose cost-effectiveness of HPV vaccines. Several key considerations merit attention: (1) To ensure accurate assessment of single-dose HPV vaccines’ effectiveness in different populations, researchers should transparently document and report detailed baseline information, such as HPV DNA status, HPV serology, sexual history, smoking status, and comorbidities. In addition, they can use multivariable analysis to account for potential differences among study populations and use multiple imputation to reduce the impact of missing data on study results. (2) Design studies specifically targeting nonavalent vaccines and high-risk populations (such as HIV patients, cancer patients, and organ transplant recipients). (3) Conduct longitudinal studies to track the effectiveness of single-dose HPV vaccines on multiple clinical outcomes in men and women, and report outcomes according to age subgroup. (4) To enhance the quality of study design, implementation, and reporting, as well as to lower the risk of systematic mistakes, adopt internationally recognized standards and guidelines, such as Strengthening the Reporting of Observational studies in Epidemiology and Consolidated Standards of Reporting Trials.

## 5. Conclusions

In summary, by incorporating a wide range of studies with robust evidence, our review supports the hypothesis that a single dose of quadrivalent and nonavalent HPV vaccines may be as effective as two or three doses in preventing vaccine-type HPV infections. However, the effectiveness of single-dose quadrivalent vaccination in preventing genital warts is weaker compared to two or three doses. To optimize the effectiveness of the HPV vaccine, it is advised to be given it at a younger age.

Further rigorous studies are needed to provide reliable evidence for single-dose HPV vaccination policies and guidelines. These studies should transparently document baseline information, conduct longitudinal tracking of the effectiveness of HPV vaccines on various clinical outcomes across different age groups, and appropriately use buffer intervals to mitigate bias while assessing vaccine effectiveness. Such research is essential to better understand the relationship between the number of vaccine doses and cross-protection against HPV, cervical cancer risk, and other clinical outcomes.

## Data Availability

The data are available in the articles included in this review.

## Supplementary Materials

The following supporting information can be downloaded at: https://www.mdpi.com/article/10.3390/vaccines12090956/s1, Table S1: search strategy in different databases; Table S2: clinical effectiveness of bivalent HPV vaccine by number of doses; Table S3: clinical effectiveness of quadrivalent HPV vaccine by number of doses; Table S4: clinical effectiveness of nonavalent HPV vaccine by number of doses.
