# Supplementary material for: The Clinical Effectiveness of Single-Dose Human Papillomavirus Vaccination
Source: Vaccines (Basel). 2024 Aug 23;12(9):956. doi: 10.3390/vaccines12090956 (PMC11436243; doi:10.3390/vaccines12090956)
Supplement: Supplementary file 1 [file vaccines-12-00956-s001.zip › vaccines-3086305-supplementary.pdf]

Supplementary Table S1. Search strategy in different databases

Database	Search method
PubMed	#1 papillomavirus vaccines[MeSH Terms] (((((((papillomavirus vaccines[Title/Abstract]) OR (papillomavirus vaccine[Title/Abstract])) OR (papillomavirus vaccination[Title/Abstract])) OR (papillomavirus vaccinations[Title/Abstract])) OR (HPV vaccines[Title/Abstract])) OR (HPV vaccine[Title/Abstract])) OR (HPV vaccinations[Title/Abstract])) OR (HPV vaccination[Title/Abstract]) 14,855<br>#2 ((((((((((((((((((vaccine effectiveness[Title/Abstract]) OR (infections[Title/Abstract])) OR (infection[Title/Abstract])) OR (HPV[Title/Abstract])) OR (cervical abnormalities[Title/Abstract])) OR (cervical abnormality[Title/Abstract])) OR (cervical neoplasms[Title/Abstract])) OR (cervical neoplasm[Title/Abstract])) OR (cervical neoplasias[Title/Abstract])) OR (cervical neoplasia[Title/Abstract])) OR (cervical intraepithelial neoplasias[Title/Abstract])) OR (cervical intraepithelial neoplasia[Title/Abstract])) OR (HPV related diseases[Title/Abstract])) OR (HPV related disease[Title/Abstract])) OR (condylomata[Title/Abstract])) OR (condyloma[Title/Abstract])) OR (genital warts[Title/Abstract])) OR (genital wart[Title/Abstract])) OR (anogenital warts[Title/Abstract])) OR (anogenital wart[Title/Abstract])) OR (anal intraepithelial neoplasia)) OR (anal intraepithelial neoplasias[Title/Abstract])1,877,922<br>#3 (((((one-dose[Title/Abstract]) OR (single-dose[Title/Abstract])) OR (two-dose[Title/Abstract])) OR (three-dose[Title/Abstract])) OR (dose[Title/Abstract])) OR (doses[Title/Abstract]) 1,552,134<br>#4 #1 AND #2 AND #3 1,988
Embase	#1 'papillomavirus vaccine'/exp OR 'papillomavirus vaccines':ti,ab,kw OR 'papillomavirus vaccine':ti,ab,kw OR 'papillomavirus vaccinations':ti,ab,kw OR 'papillomavirus vaccination':ti,ab,kw OR 'hpv vaccines':ti,ab,kw OR 'hpv vaccine':ti,ab,kw OR 'hpv vaccinations':ti,ab,kw OR 'hpv vaccination':ti,ab,kw 23559<br>#2 'vaccine effectiveness':ti,ab,kw OR 'infections':ti,ab,kw OR 'infection':ti,ab,kw OR 'hpv':ti,ab,kw OR 'cervical abnormalities':ti,ab,kw OR 'cervical abnormality':ti,ab,kw OR 'cervical neoplasms':ti,ab,kw OR 'cervical neoplasm':ti,ab,kw OR 'cervical neoplasias':ti,ab,kw OR 'cervical neoplasia':ti,ab,kw OR 'cervical intraepithelial neoplasias':ti,ab,kw OR 'cervical intraepithelial neoplasia':ti,ab,kw OR 'hpv related diseases':ti,ab,kw OR 'hpv related disease':ti,ab,kw OR 'condylomata':ti,ab,kw OR 'condyloma':ti,ab,kw OR 'genital warts':ti,ab,kw OR 'genital wart':ti,ab,kw OR 'anogenital warts':ti,ab,kw OR 'anogenital wart':ti,ab,kw 2,449,038<br>#3 'one-dose':ti,ab,kw OR 'single-dose':ti,ab,kw OR 'two-dose':ti,ab,kw OR 'three-dose':ti,ab,kw OR 'dose':ti,ab,kw OR 'doses':ti,ab,kw 2,270,282<br>#4 #1 AND #2 AND #2,733
Cochrane	#1 MeSH descriptor: [Papillomavirus Vaccines] explode all trees 736<br>#2 ('papillomavirus vaccine' OR 'papillomavirus vaccines' OR 'papillomavirus vaccine' OR 'papillomavirus vaccinations' OR 'papillomavirus vaccination' OR 'hpv vaccines' OR 'hpv vaccine' OR 'hpv vaccinations' OR 'hpv vaccination'):ti,ab,kw 1,801<br>#3 ('vaccine effectiveness'OR 'infections' OR 'infection' OR 'hpv' OR 'cervical abnormalities' OR 'cervical abnormality' OR 'cervical neoplasms' OR 'cervical neoplasm' OR 'cervical neoplasias' OR 'cervical neoplasia' OR 'cervical intraepithelial neoplasias' OR 'cervical intraepithelial neoplasia' OR 'hpv related diseases' OR ''hpv related disease' OR 'condylomata' OR 'condyloma' OR 'genital warts' OR 'genital wart' OR 'anogenital warts' OR 'anogenital wart'):ti,ab,kw 118,007<br>#4 ('one-dose' OR 'single-dose' OR 'two-dose' OR 'three-dose' OR 'dose' OR 'doses'):ti,ab,kw 385,660<br>#5 #1 OR #2 1801<br>#6 #3 AND #4 AND #5 665

Our selection of databases and search terms was informed by reviews to comprehensively cover the relevant literature on the clinical effectiveness of single-dose HPV vaccination.

We chose PubMed, Cochrane Library, and EMBASE for their comprehensive coverage and relevance to medical research: 1) PubMed is one of the most widely used databases in the medical field. 2) Cochrane Library is renowned for its high-quality evidence-based reviews and clinical trials. 3) EMBASE provides extensive coverage of biomedical and pharmacological literature. It includes many journals not indexed in PubMed.

Selection of several terms mentioned above for the following reasons: 1) We chose ''papillomavirus vaccine'', ''papillomavirus vaccination'', ''HPV vaccine'', and ''HPV vaccination'' to capture all literature specifically related to HPV vaccines. 2) We chose terms "vaccine effectiveness", ''infection'', ''HPV'', "cervical abnormality", ''cervical neoplasm'', ''cervical neoplasia'', ''cervical intraepithelial neoplasia'', ''HPV related disease'', "condyloma", ''genital wart'', "anogenital wart'', and "anal intraepithelial neoplasia" since they cover the wide spectrum of potential clinical outcomes that HPV vaccination can prevent. 3) We chose "single-dose", ''one-dose'', ''two-dose'', "three-dose", and ''dose'' to capture studies on various HPV vaccination dosage schedules. This approach ensured that we included certain studies which were not primarily focusing on examining the effects of HPV vaccines administered as single dose, but contained significant information regarding the efficacy of single-dose vaccination.

Supplementary Table S2. Clinical effectiveness of Bivalent HPV vaccine by number of doses

Endpoint/ Vaccine/ Study	Outcome	Adjustment	RR or PR (95%CI), p value		
			3 doses/control	2 doses/control	1 dose/control
**Cervical HPV infection in women**					
Kreimer 2011 [13]	Persistent HPV 16/18 infection for 6 months in 4-year follow-up	— —	RR = 0.164 (0.115-0.231)	RR = 0.188 (0.064-0.532)	RR = 0 (0-0.212)
	Persistent HPV 16/18 infection for 12 months in 4-year follow-up		RR = 0.191 (0.123-0.289)	RR = 0.159 (0.037-0.498)	RR = 0 (0-0.335)
Kavanagh 2014 [17]	HPV 16/18	Birth cohort year, deprivation score	aOR = 0.43 (0.34–0.55)	aOR = 0.68 (0.42–1.12)	aOR = 0.95 (0.51–1.76)
	HPV 31/33/45		aOR = 0.53 (0.38–0.74)	aOR = 0.55 (0.26–1.17)	aOR = 1.44 (0.70–2.96)
	HPV 35/39/51/52/56/58/59		aOR = 0.81 (0.66–0.99)	aOR = 0.90 (0.59–1.39)	aOR = 1.09 (0.62–1.91)
	Any HPV		aOR = 0.66 (0.54–0.80)	aOR = 0.88 (0.58–1.33)	aOR = 1.18 (0.66–2.11)
Kreimer 2015 [21]	HPV 16/18	— —	RR = 0.230 (0.209-0.253)	RR = 0.240 (0.147-0.380)	RR = 0.143 (0.063-0.293)
	Persistent HPV 16/18 infection for 6 months in 4-year follow-up		RR = 0.109 (0.090-0.132)	RR = 0.103 (0.031-0.267)	RR = 0.034 (0.002-0.183)

	Persistent HPV 16/18 infection for 12 months in 4-year follow-up		RR = 0.130 (0.103-0.163)	RR = 0.104 (0.025-0.311)	RR = 0.049 (0.002-0.268)
	HPV 31/33/45		RR = 0.403 (0.370-0.440)	RR = 0.623 (0.441-0.876)	RR = 0.634 (0.378-1.054)
	Persistent HPV 31/33/45 infection for 6 months in 4-year follow-up		RR = 0.399 (0.346-0.460)	RR = 0.693 (0.370-1.279)	RR = 0.512 (0.215-1.169)
	Persistent HPV 31/33/45 infection for 12 months		RR = 0.451 (0.377-0.538)	RR = 0.924 (0.392-2.178)	RR = 0.539 (0.160-0.332)
Cuschieri 2016 [22]	HPV 16/18	Age at first vaccination, SMID	aOR = 0.27 (0.20–0.36)	aOR = 0.45 (0.29-0.69)	aOR = 0.52 (0.31-0.83)
	HPV 31/33/45		aOR =0.45 (0.30-0.67)	aOR = 0.52 (0.28–0.92)	aOR = 1.16 (0.55–1.85)
Kavanagh 2017 [27]	HPV 16/18	Birth cohort, number of doses received, Scottish Index of Multiple Deprivation (SMID) quintile	aOR = 0.40 (0.33–0.48)	aOR = 0.75 (0.57–0.99)	aOR = 0.89 (0.63–1.25)
	HPV 31/33/45		aOR = 0.46 (0.36–0.58)	aOR = 0.64 (0.42–0.93)	aOR = 1.10 (0.71–1.65)
	HPV 35/39/51/52/56/58/59		aOR = 0.96 (0.83–1.10)	aOR = 1.11 (0.88–1.40)	aOR = 1.06 (0.79–1.42)
	Any HPV		aOR = 0.79 (0.69–0.90)	aOR = 1.06 (0.84–1.33)	aOR = 1.10 (0.82–1.47)
Safaeian 2018 [32]	HPV 16/18 infections at years 7	——	*HPV infection rate(%):*<br>Case: 1.0 (0.6-1.5)<br>Control: 6.6 (5.7-7.7)	*HPV infection rate(%):*<br>0,6:<br>Case: 1.3 (0.1-6.1)<br>Control: 6.6 (5.7 to 7.7)<br>0,1:<br>Case: 1.0 (0.2-3.4)<br>Control:6.6 (5.7-7.7)	*HPV infection rate(%):*<br>Case: 0.0 (0.0-2.2)<br>Control: 6.6 (5.7-7.7)
	HPV 31/33/45 infections at years 7		*HPV infection rate(%):*<br>Case: 2.3 (1.8-3.1)<br>Control: 5.5 (4.7-6.5)	*HPV infection rate(%):*<br>0,6:<br>Case: 0.0 (0.0-3.7)<br>Control: 5.5 (4.7-6.5)<br>0,1:<br>Case: 2.1 (0.7-4.9)<br>Control: 5.5 (4.7-6.5)	*HPV infection rate(%):*<br>Case: 1.5 (0.3-4.8)<br>Control: 5.5 (4.7-6.5)
	HPV 35/39/51/52/56/58/59 at years 7		*HPV infection rate(%):*<br>Case: 15.2 (13.7-16.8)<br>Control: 13.0 (11.7-14.4)	*HPV infection rate(%):*<br>0,6:<br>Case: 13.9 (7.5-22.9)<br>Control: 13.0 (11.7-14.4)<br>0,1:<br>Case: 14.5 (10.1-20.0)<br>Control: 13.0 (11.7-14.4)	*HPV infection rate(%):*<br>Case: 13.4 (8.4-20.0)<br>Control: 13.0 (11.7-14.4)

	Non-carcinogenic HPV at years 7		*HPV infection rate(%):* Case: 14.5 (13.1-16.1) Control: 13.4 (12.1-14.9)	*HPV infection rate(%):* 0,6: Case: 13.9 (7.5-22.9) Control: 13.4 (12.1-14.9) 0,1: Case: 15.5 (10.9-21.2) Control: 13.4 (12.1-14.9)	*HPV infection rate(%):* Case: 11.9 (7.2-18.3) Control: 13.4 (12.1-14.9)
Kreimer 2020 [43]	HPV 16/18 infections at year 11	— —	RR = 0.151 (0.068-0.302)	RR = 0.416 (0.021-2.109)	RR = 0.461 (0.076-1.571)
	HPV 35/39/51/52/56/58/59 at year 11		RR = 0.937 (0.764-1.161)	RR = 0.871 (0.349-1.841)	RR = 1.286 (0.748-2.091)
	Non-carcinogenic HPV at year 11		RR = 1.080 (0.950-1.228)	RR = 0.783 (0.443-1.294)	RR = 1.363 (0.991-1.837)
	HPV 16/18 at years 9 or 11		RR = 0.198 (0.130-0.293)	RR = 0.162 (0.008-0.805)	RR = 0.179 (0.003-0.598)
	HPV 35/39/51/52/56/58/59 at years 9 or 11		RR = 1.039 (0.901-1.199)	RR = 1.283 (0.788-1.988)	RR = 0.934 (0.612-1.376)
	Non-carcinogenic HPV at years 9 or 11		RR = 1.050 (0.952-1.158)	RR = 1.185 (0.841-1.628)	RR = 1.205 (0.936-1.533)
Barnabas 2022 [50]	Persistent HPV 16/18 for ≥4 months in 18-month follow-up	— —	— —	— —	HR = 0.025 (0.003-0.184)
Barnabas 2023 [51]	Persistent HPV 16/18 for ≥4 months in 3-year follow-up	— —	— —	— —	HR = 0.035 (0.006-0.100)
	Persistent HPV 31/33/45 for ≥4 months in 3-year follow-up		— —	— —	HR = 0.899 (0.583-1.387)
**Cervical histological and cytological abnormalities in women**					
Pollock 2014 [18]	CIN3	Cohort year, SMID and age in months	aOR = 0.45 (0.35–0.58)	aOR = 0.77 (0.49–1.21)	aOR = 1.42 (0.89–2.28)
	CIN2		aOR = 0.50 (0.40–0.63)	aOR = 0.81 (0.54–1.22)	aOR = 1.03 (0.62–1.71)
	CIN1		aOR = 0.71 (0.58–0.87)	aOR = 0.65 (0.42–1.01)	aOR = 0.98 (0.59–1.63)

Cameron 2017 [26]	CIN3	– –	*Crude incidence rate*<br>*Per 100000 person-years:*<br>SMID 1:<br>Case: 6.9 (2.2 to 16.1)<br>Control: 14.5 (12.7 to 16.4)<br>SMID 2:<br>Case: 17.4 (8 to 33)<br>Control: 13.2 (11.4 to 15.1)<br>SMID 3:<br>Case: 16 (5.9 to 34.8)<br>Control: 9.4 (7.8 to 11.2)<br>SMID 4:<br>Case: 3.6 (0.1 to 20)<br>Control: 8.3 (6.8 to 10.1)<br>SMID 5:<br>Case: 4 (0.1-22)<br>Control: 5.1 (4-6.5)	*Crude incidence rate*<br>*Per 100000 person-years:*<br>SMID 1:<br>Case: 7.7 (3.7 to 14.2)<br>Control: 14.5 (12.7 to 16.4)<br>SMID 2:<br>Case: 7.1 (2.9 to 14.7)<br>Control: 13.2 (11.4 to 15.1)<br>SMID 3:<br>Case: 11.2 (5.1 to 21.3)<br>Control: 9.4 (7.8 to 11.2)<br>SMID 4:<br>Case: 3.1 (0.4 to 11.1)<br>Control: 8.3 (6.8 to 10.1)<br>SMID 5:<br>Case: 7.4 (2-18.9)<br>Control: 5.1 (4-6.5)	*Crude incidence rate*<br>*Per 100000 person-years:*<br>SMID 1:<br>Case: 3.3 (2.3 to 4.7)<br>Control: 14.5 (12.7 to 16.4)<br>SMID 2:<br>Case: 3.9 (2.8 to 5.3)<br>Control: 13.2 (11.4 to 15.1)<br>SMID 3:<br>Case: 3.5 (2.5 to 4.8)<br>Control: 9.4 (7.8 to 11.2)<br>SMID 4:<br>Case: 2.9 (2 to 4.2)<br>Control: 8.3 (6.8 to 10.1)<br>SMID 5:<br>Case: 2.5 (1.7-3.6)<br>Control: 5.1 (4-6.5)
	CIN2		*Crude incidence rate*<br>*Per 100000 person-years:*<br>SMID 1:<br>Case: 20.6 (11.5 to 34) Control:<br>17.6 (15.6-19.7)<br>SMID 2:<br>Case: 1.9 (0.1 to 10.8)<br>Control: 13.9 (12.1-15.9)<br>SMID 3:<br>Case: 18.6 (7.5 to 38.2)<br>Control: 9.5 (7.9 to 11.3)<br>SMID 4:<br>Case: 14.3 (3.9 to 36.7)<br>Control: 11.1 (9.3 to 13.1)<br>SMID 5:<br>Case: 0<br>Control: 8.3 (6.9-9.9)	*Crude incidence rate*<br>*Per 100000 person-years:*<br>SMID 1:<br>Case: 8.5 (4.2 to 15.2)<br>Control: 17.6 (15.6-19.7)<br>SMID 2:<br>Case: 20.3 (12.4 to 31.3)<br>Control: 13.9 (12.1-15.9)<br>SMID 3:<br>Case: 6.2 (2.1 to 14.6)<br>Control: 9.5 (7.9 to 11.3)<br>SMID 4:<br>Case: 7.7 (2.5 to 18)<br>Control: 11.1 (9.3 to 13.1)<br>SMID 5:<br>Case: 5.5(1.1-16.2)<br>Control: 8.3 (6.9-9.9)	*Crude incidence rate*<br>*Per 100000 person-years:*<br>SMID 1:<br>Case: 6.4 (4.9 to 8.2)<br>Control: 7.6 (15.6-19.7)<br>SMID 2:<br>Case: 5.5 (4.2 to 7.1)<br>Control: 13.9 (12.1-15.9)<br>SMID 3:<br>Case: 5.5 (4.2 to 7)<br>Control: 9.5 (7.9 to 11.3)<br>SMID 4:<br>Case: 4.2 (3 to 5.7)<br>Control: 11.1 (9.3 to 13.1)<br>SMID 5:<br>Case: 2.6 (1.7-3.7)<br>Control: 8.3 (6.9-9.9)
	CIN1		aRR = 0.825 (0.695-0.979)	aRR = 1.031 (0.744-1.428)	aRR = 0.752 (0.453-1.248)

Palmer 2019 [38]	CIN3+	Deprivation, ruarality	*Age at the first dose:*<br>12-13:<br>aOR = 0.14 (0.08-0.25)<br>14:<br>aOR = 0.18 (0.07-0.43)<br>15:<br>aOR = 0.29(0.19-0.44)<br>16:<br>aOR = 0.27(0.18-0.41)<br>17:<br>aOR = 0.55(0.36-0.83)<br>≥18:<br>aOR = 0.85(0.52-1.37)	*Age at the first dose:*<br>all ages:<br>aOR = 0.77 (0.48–1.24)	*Age at the first dose:*<br>all ages:<br>aOR = 1.19 (0.70–2.05)
	CIN2		*Age at the first dose:*<br>12-13:<br>aOR = 0.11(0.06-0.19)<br>14:<br>aOR = 0.13(0.05-1.31)<br>15:<br>aOR = 0.35(0.25-0.48)<br>16:<br>aOR = 0.31(0.23-0.44)<br>17:<br>aOR = 0.45(0.30-0.66)<br>≥18:<br>aOR = 0.75(0.48-1.15)	*Age at the first dose:*<br>all ages:<br>aOR = 0.70 (0.45–1.07)	*Age at the first dose:*<br>all ages:<br>aOR = 0.95 (0.56–1.59)
	CIN1		*Age at the first dose:*<br>12-13:<br>aOR = 0.22(0.14-0.34)<br>14:<br>aOR = 0.48(0.29-0.79)<br>15:<br>aOR = 0.48(0.36-0.65)<br>16:<br>aOR = 0.58(0.44-0.77)<br>17:<br>aOR = 0.59(0.41-0.86)<br>≥18:<br>aOR = 0.79(0.50-1.25)	*Age at the first dose:*<br>all ages:<br>aOR = 0.70 (0.43–1.12)	*Age at the first dose:*<br>all ages:<br>aOR = 0.94 (0.53–1.68)

HSIL-H	*Age at the first dose:*<br>12-13:<br>aOR = 0.07(0.03-0.14)<br>14:<br>aOR = 0.23(0.11-0.46)<br>15:<br>aOR = 0.28(0.20-0.41)<br>16:<br>aOR = 0.25(0.17-0.37)<br>17:<br>aOR = 0.42(0.27-0.63)<br>≥18:<br>aOR = 0.88(0.58-1.35)	*Age at the first dose:*<br>all ages:<br>aOR = 0.62 (0.38–1.00)	*Age at the first dose:*<br>all ages:<br>aOR = 1.02 (0.59–1.75)
HSIL-M	*Age at the first dose:*<br>12-13:<br>aOR = 0.20 (0.15-0.28)<br>14:<br>aOR = 0.26 (0.16-0.42)<br>15:<br>aOR = 0.36 (0.28-0.47)<br>16:<br>aOR = 0.35 (0.27-0.45)<br>17:<br>aOR = 0.60 (0.46-0.78)<br>≥18:<br>aOR=0.65 (0.45-0.93)	*Age at the first dose:*<br>all ages:<br>aOR = 0.91 (0.67–1.24)	*Age at the first dose:*<br>all ages:<br>aOR = 0.89 (0.58–1.37)
LSIL	*Age at the first dose:*<br>12-13:<br>aOR = 1.38 (1.28-1.48)<br>14:<br>aOR = 1.37 (1.22-1.53)<br>15:<br>aOR = 1.31 (1.22-1.41)<br>16:<br>aOR = 1.05 (0.97-1.13)<br>17:<br>aOR = 0.77 (0.86-0.96)<br>≥18:<br>aOR = 0.80 (0.68-0.94)	*Age at the first dose:*<br>all ages:<br>aOR = 1.09 (0.94–1.26)	*Age at the first dose:*<br>all ages:<br>aOR = 1.27 (1.05–1.53)
ASCUS	*Age at the first dose:*<br>12-13:<br>aOR = 0.58(0.54-0.62)<br>14:	*Age at the first dose:*<br>all ages:<br>aOR = 0.94 (0.85–1.04)	*Age at the first dose:*<br>all ages:<br>aOR = 0.94 (0.81–1.08)

			aOR = 0.55(0.50-0.62)<br>15:<br>aOR = 0.59(0.50-0.63)<br>16:<br>aOR = 0.75(0.71-0.80)<br>17:<br>aOR = 0.86(0.80-0.93)<br>≥18:<br>aOR = 0.98(0.89-1.08)		
Acuti Martellucci 2021 [47]	HSIL	Year of birth, being born abroad, residential area, number of screens, test kit	— —	— —	aOR = 1.19 (0.28-5.03)
	LSIL		aOR = 0.38 (0.05-2.81)	aOR = 0.63 (0.25-1.57)	aOR = 0.57 (0.31-1.03)
**Genital warts in women**					
Navarro-Illana 2017 [28]	Genital warts	Age, calendar year, health department	aRR = 1.14 (0.61–1.95)	aRR = 2.25 (0.98–4.21)	aRR = 1.09 (0.21–2.7)

Abbreviations: HPV, human papillomavirus; SMID, Scottish index of multiple deprivation; aRR, adjusted risk ratios; aHR, adjusted hazard ratios; aIRR, adjusted incidence rate ratios; aPR, adjusted prevalence ratios; aOR, adjusted odds ratios; CIN, cervical intraepithelial neoplasia; AIS, adenocarcinoma in situ; HSIL, high-grade squamous intraepithelial lesions (HSIL-M=mild dyskaryosis; HSIL-H=high grade dyskaryosis); LSIL, low-grade squamous intraepithelial lesions; ASCH, atypical squamous cells, cannot exclude HSIL; ASC-US, atypical squamous cells of undetermined significance.

Supplementary Table S3. Clinical effectiveness of Quadrivalent HPV vaccine by number of doses

Endpoint/ Vaccine/ Study	Outcome	Adjustment	RR or PR (95%CI), p value		
			**3 doses/control**	**2 doses/control**	**1 dose/control**
**Cervical HPV infection in women**					
Sankaranarayanan 2016 [25]	HPV 16	——	*HPV infection rate (%):* 0.4 (0.0–1.3)	*HPV infection rate (%):* days 1, ≥180: 0.6 (0.1–1.7) days 1, 60: 1.1 (0.5–2.2)	*HPV infection rate (%):* 1.0 (0.5–2.0)
	HPV 18		——	*HPV infection rate (%):* days 1, ≥180: 0.2 (0.0–1.1) days 1, 60: 0.1 (0.0–0.8)	*HPV infection rate (%):* 0.1 (0.0–0.6)
	HPV 16/18		*HPV infection rate (%):* 0.4 (0.0–1.3)	*HPV infection rate (%):* days 1, ≥180: 0.8 (0.2–1.9) days 1, 60: 1.3 (0.6–2.4)	*HPV infection rate (%):* 1.1 (0.6–2.1)
	HPV 6/11		*HPV infection rate (%):* 0.2 (0.0–1.0)	*HPV infection rate (%):* days 1, ≥180: 0.2 (0.0–1.1) days 1, 60: 0.7 (0.2–1.6)	*HPV infection rate (%):* 0.5 (0.1–1.2)
	HPV 6/11/16/18		*HPV infection rate (%):* 0.6 (0.1–1.6)	*HPV infection rate (%):* days 1, ≥180: 1.0 (0.3–2.2) days 1, 60: 2.0 (1.1–3.3)	*HPV infection rate (%):* 1.6 (0.9–2.7)
	HPV 31/33/45		*HPV infection rate (%):* 6.0 (4.1–8.3)	*HPV infection rate (%):* days 1, ≥180: 4.9 (3.3–7.2) days 1, 60: 4.6 (3.2–6.4)	*HPV infection rate (%):* 8.9 (7.0–10.9)
	Non-HPV 6/11/16/18/31/33/45		*HPV infection rate (%):* 13·8% (11.0–17.0)	*HPV infection rate (%):* days 1, ≥180: 9.1 (6.8–11.9) days 1, 60: 9.5 (7.4–11.9)	*HPV infection rate (%):* 13.6 (11.4–16.0)
	Persistent HPV 16 infection for ≥12 months in 4-year follow-up		*HPV infection rate (%):* 0	*HPV infection rate (%):* days 1, ≥180: 0 days 1, 60: 0	*HPV infection rate (%):* 0
	Persistent HPV 18 infection for ≥12 months in 4-year follow-up		*HPV infection rate (%):* 0	*HPV infection rate (%):* days 1, ≥180: 0 days 1, 60: 0	*HPV infection rate (%):* 0

	Persistent HPV 16/18 infection for ≥12 months in 4-year follow-up		*HPV infection rate (%):* 0	*HPV infection rate (%):* days 1, ≥180: 0 days 1, 60: 0	*HPV infection rate (%):* 0
	Persistent HPV 6/11 infection for ≥12 months in 4-year follow-up		*HPV infection rate (%):* 0	*HPV infection rate (%):* days 1, ≥180: 0 days 1, 60: 0.1	*HPV infection rate (%):* 0
	Persistent HPV 6/11/16/18 infection for ≥12 months in 4-year follow-up		*HPV infection rate (%):* 0	*HPV infection rate (%):* days 1, ≥180: 0 days 1, 60: 0.1	*HPV infection rate (%):* 0
	Persistent HPV 31/33/45 infection for ≥12 months in 4-year follow-up		*HPV infection rate (%):* 0	*HPV infection rate (%):* days 1, ≥180: 0 days 1, 60: 0.3	*HPV infection rate (%):* 0.5
	Persistent non-HPV 6/11/16/18/31/33/45 infection for ≥12 months in 4-year follow-up		*HPV infection rate (%):* 0.7	*HPV infection rate (%):* days 1, ≥180: 0.9 days 1, 60: 0.6	*HPV infection rate (%):* 0.5
Sankaranarayanan 2018 [33]	Persistent HPV 16/18 infection for ≥12 months in 7-year follow-up	— —	*HPV infection rate (%):* Case: 0.2 (0.0-0.9) Control: 1.2 (0.7-2.1)	*HPV infection rate (%):* days 1, ≥180: Case: 0 Control: 1.2 (0.7-2.1) days 1, 60: Case: 0.4 (0.1-1.1) Control: 1.2 (0.7-2.1)	*HPV infection rate (%):* Case: 0 (0-0) Control: 1.2 (0.7-2.1)
	Persistent HPV 31/33/45 infection for ≥12 months in 7-year follow-up		*HPV infection rate (%):* Case: 0.2 (0.0-0.9) Control: 0.5 (0.2-1.1)	*HPV infection rate (%):* days 1, ≥180: Case: 0.2 (0.0-0.9) Control: 0.5(0.2-1.1) days 1, 60: Case: 0.2(0.0-0.9) Control: 0.5(0.2-1.1)	*HPV infection rate (%):* Case: 0.7 (0.3-1.5) Control: 0.5 (0.2-1.1)

	Persistent non-vaccine targeted HPV for ≥12 months in 7-year follow-up		*HPV infection rate (%):*<br>Case: 2.6 (1.5-4.3)<br>Control: 2.3 (1.5-3.3)	*HPV infection rate (%):*<br>days 1, ≥180:<br>Case: 1.0 (0.4-2.1)<br>Control: 2.3 (1.5-3.3)<br>days 1, 60:<br>Case: 1.1 (0.5-2.1)<br>Control: 2.3 (1.5-3.3)	*HPV infection rate (%):*<br>Case: 1.7 (1.1-2.7)<br>Control: 2.3 (1.5-3.3)
Sonawane 2019 [39]	HPV 6/11/16/18	Variables in the model, age as a linear term	aPD = -4.3 (-4.6, -4.0)	aPD = -1.7 (-2.4, -0.1)	aPD = -5.0 (-5.6, -4.5)
	HPV 31/33/45		*HPV infection rate (%):*<br>Case: 6.3 (3.2-9.4)<br>Control: 5.4 (3.7-7.1)	*HPV infection rate (%):*<br>Case: 2.8 (0.0-6.0)<br>Control 5.4 (3.7-7.1)	*HPV infection rate (%):*<br>Case: 10.7 (3.5-18.0)<br>Control: 5.4 (3.7-7.1)
	HPV 35/39/51/52/56/58/59/68		*HPV infection rate (%):*<br>Case: 27.2 (20.1-33.5)<br>Control: 25.2 (21.6-28.8)	*HPV infection rate (%):*<br>Case: 28.1 (17.8-38.4)<br>Control: 25.2 (21.6-28.8)	*HPV infection rate (%):*<br>Case: 22.7 (12.4-32.9)<br>Control: 25.2 (21.6-28.8)
Markowitz 2020 [44]	HPV 6/11/16/18	— —	*Age at the first dose:*<br>All ages:<br>aPR = 0.14 (0.09–0.22)<br>≤18:<br>aPR = 0.06 (0.04–0.12)<br>>18:<br>aPR = 0.77 (0.44–1.36)	*Age at the first dose:*<br>All ages:<br>aPR = 0.13 (0.04–0.43)<br>≤18:<br>aPR = 0.05 (0.01–0.39)<br>>18:<br>aPR = 0.36 (0.09–1.44)	*Age at the first dose:*<br>All ages:<br>aPR = 0.22 (0.09–0.55)<br>≤18:<br>aPR = 0.06 (0.01–0.42)<br>>18:<br>aPR = 0.57 (0.21–1.53)
	non-HPV 6/11/16/18		PR =1.18 (1.07–1.30)	PR =1.30 (1.11–1.51)	PR =1.17 (1.00–1.38)
	HPV 16/18/31/33/35/39/45 /51/52/56/58/59/66/68		PR =1.07 (0.94–1.21)	PR =1.31 (1.08–1.60)	PR =1.04 (0.84–1.30)
	HPV 31/33/45		PR = 0.68 (0.46–1.01)	PR = 1.42 (0.80–2.50)	PR = 1.69 (0.99–2.88)
Batmunkh 2020 [41]	HPV 16	Employment status, income	— —	— —	aPR = 0.10 (0.01–0.73)
	HPV 18		— —	— —	aPR = 0.11 (0.00–0.70)

	Any HPV		— —	— —	aPR = 0.67 (0.47–0.95)
	HPV 16/18		— —	— —	aPR = 0.08 (0.01–0.56)
	HPV 35/39/51/52/56/58/59/68		— —	— —	aPR = 0.83 (0.56–1.23)
	HPV 16 + other type / 18 + other type / 16 + 18 + other type		— —	— —	aPR = 0.11 (0.00–0.64)
Basu 2021 [49]	HPV 16/18	Study site, birth cohort at recruitment, religion, total number of pregnancies, age, birth cohort at recruitment	aHR = 0.336 (0.237-0.464)	aHR = 0.323 (0.228-0.448)	aHR = 0.365 (0.269-0.488)
	HPV 6/11/16/18		aHR = 0.453 (0.350-0.591)	aHR = 0.410 (0.309-0.531)	aHR = 0.459 (0.359-0.582)
	HPV 31/33/45		aHR = 0.454 (0.334-0.617)	aHR = 0.460 (0.335-0.615)	aHR = 0.565 (0.435-0.746)
	Any HPV		aHR = 0.698 (0.606-0.803)	aHR = 0.655 (0.569-0.755)	aHR = 0.698 (0.615-0.799)
	Persistent HPV 16/18 infection for ≥10 months in 10-year follow-up		aHR = 0.067 (0.003-0225)	aHR = 0.069 (0.002-0.227)	aHR = 0.046 (0.001-0.150)
	Persistent HPV 6/11/16/18 infection for ≥10 months in 10-year follow-up		aHR = 0.097 (0.015-0.281)	aHR = 0.063 (0.002-0.202)	aHR = 0.066 (0.009-0.189)
	Persistent HPV 31/33/45 infection for ≥10 months in 10-year follow-up		aHR = 0.612 (0.198-2.244)	aHR=0.916 (0.343-3.393)	aHR = 0.912 (0.374-3.308)
	Persiestant any HPV infection for ≥10 months in 10-year follow-up		aHR = 0.607(0.398-0.932)	aHR = 0.633 (0.421-0.984)	aHR = 0.646 (0.44-0.963)
Reyburn 2023 [53]	HPV 16/18/31/33/35/39/45/51/52/56/58/59/66/68	Age, ethnicity and smoking	*HPV infection rate (%):* Case: 41.5 Control: 39.4	*HPV infection rate (%):* Case: 40.4 Control: 39.4	*HPV infection rate (%):* Case: 37.8 Control: 39.4
	HPV 16/18		aPR =0.11 (0.04, 0.36)	aPR =0.000062 (0.000046, 0.000083)	aPR = 0.19 (0.07, 0.52)
	HPV 6/11		*HPV infection rate (%):* Case: 0.5 Control: 2.4	*HPV infection rate (%):* Case: 1.3 Control: 2.4	*HPV infection rate (%):* Case: 0 Control: 2.4

	HPV 31/33/35/39/45/ 51/52/56/58/59/66/68		aPR = 1.10 (0.88, 1.38)	aPR = 1.16 (0.88, 1.52)	aPR = 1.09 (0.85, 1.40)
	HPV 6/11/16/18/31/33/45/52/58		*HPV infection rate (%):*<br>Case: 12.2<br>Control: 22.6	*HPV infection rate (%):*<br>Case: 9.1<br>Control: 22.6	*HPV infection rate (%):*<br>Case: 25.8<br>Control: 22.6
	HPV 6/11/31/33/45/52/58		*HPV infection rate (%):*<br>Case: 10.6<br>Control: 12.8	*HPV infection rate (%):*<br>Case: 9.1<br>Control: 12.8	*HPV infection rate (%):*<br>Case: 19.7<br>Control: 12.8
**Penile, scrotal or anal HPV infection in men**					
Widdice 2019<br>[40]	HPV 6/11/16/18	— —	*HPV infection rate (%):*<br>Case: 22<br>Control: 25	*HPV infection rate (%):*<br>Case: 27<br>Control: 25	*HPV infection rate (%):*<br>Case: 19<br>Control: 25
**Oral HPV infection in women**					
	HPV 16		*HPV infection rate (%):*<br>Case: 1.5<br>Control: 3.3	*HPV infection rate (%):*<br>0,180:<br>Case: 1.2<br>Control: 3.3<br>0,60:<br>Case: 0<br>Control: 3.3	*HPV infection rate (%):*<br>Case: 3.5<br>Control: 3.3
Gheit 2023<br>[52]	HPV 18	Age	*HPV infection rate (%):*<br>Case: 0.6<br>Control: 0.8	*HPV infection rate (%):*<br>0,180:<br>Case: 0<br>Control: 0.8<br>0,60: 1<br>Case: 0<br>Control: 0.8	*HPV infection rate (%):*<br>Case: 0<br>Control: 0.8
	HPV 16/18		*HPV infection rate (%):*<br>Case: 2.1<br>Control: 4.2	*HPV infection rate (%):*<br>0,180:<br>Case: 1.2<br>Control: 4.2<br>0,60: 1.1 vs. 4.2	*HPV infection rate (%):*<br>Case: 3.5<br>Control: 4.2

	HPV 6/11/16/18		*HPV infection rate (%):*<br>Case: 4.3<br>Control: 7.5	*HPV infection rate (%):*<br>0,180:<br>Case: 3.2<br>Control: 7.5<br>0,60:<br>Case: 3.7<br>Control: 7.5	*HPV infection rate (%):*<br>Case: 8.2<br>Control: 7.5
	Non-HPV 6/11/16/18		*HPV infection rate (%):*<br>Case: 1.0<br>Control: 2.1	*HPV infection rate (%):*<br>0,180:<br>Case: 2.5<br>Control: 2.1<br>0,60:<br>Case: 0<br>Control: 2.1	*HPV infection rate (%):*<br>Case: 4.7<br>Control: 2.1
	Any HPV		*HPV infection rate (%):*<br>Case: 4.7<br>Control: 8.7	*HPV infection rate (%):*<br>0,180:<br>Case: 5.7<br>Control: 8.7<br>0,60:<br>Case: 3.7<br>Control: 8.7	*HPV infection rate (%):*<br>Case: 11.7<br>Control: 8.7
**Cervical histological and cytological abnormalities in women**					
Gertig 2013<br>[14]	CIN3/AIS	— —	HR = 0.53 (0.36–0.77)	HR = 0.87 (0.46–1.67)	HR = 1.40 (0.75–2.61)
	CIN2+/AIS		HR = 0.61 (0.48–0.78)	HR = 1.02 (0.68–1.53)	HR = 1.47 (0.97–2.23)
	CIN2		HR = 0.70 (0.52-0.94)	HR = 0.99 (0.59-1.64)	HR = 1.29 (0.76-2.20)
	CIN1		HR = 0.82 (0.66 -1.01)	HR = 0.90 (0.61-1.33)	HR = 0.89 (0.56-1.41)
	High-grade		HR = 0.71 (0.61-0.83)	HR = 0.95 (0.73-1.23)	HR = 0.85 (0.62-1.17)
	Low-grade		HR = 0.79 (0.75-0.84)	HR = 0.64 (0.57-0.72)	HR = 0.67 (0.59-0.76)

Crowe 2014<br>[15]	CIN2+/AIS	Socioeconomic status,<br>remoteness, year of of birth,<br>quartile of follow-up times	*Age at the first dose:*<br>all ages:<br>aOR = 0.54 (0.43–0.67)<br>11-14:<br>aOR = 0.71 (0.19-2.66)<br>15-18:<br>aOR = 0.43 (0.31-0.62)<br>19-22:<br>aOR = 0.47(0.32-0.70)<br>23-27:<br>aOR = 0.95 (0.63-1.45)	*Age at the first dose:*<br>all ages:<br>aOR = 0.79 (0.64–0.98)<br>11-14:<br>— —<br>15-18:<br>aOR = 0.77(0.51-1.16)<br>19-22:<br>aOR = 0.68(0.49-0.94)<br>23-27:<br>aOR = 0.97(0.65-1.45)	*Age at the first dose:*<br>all ages:<br>aOR = 0.95 (0.77–1.16)<br>11-14:<br>aOR = 2.54 (0.54-11.8)<br>15-18:<br>aOR = 0.86 (0.54-1.37)<br>19-22:<br>aOR = 0.75 (0.55-1.02)<br>23-27:<br>aOR = 1.22 (0.89-1.70)
	other cases		*Age at the first dose:*<br>all ages:<br>aOR = 0.66 (0.62–0.70)<br>11-14:<br>aOR =0.60 (0.45-0.80)<br>15-18:<br>aOR = 0.64 (0.59-0.69)<br>19-22:<br>aOR = 0.70 (0.61-0.79)<br>23-27:<br>aOR = 0.72 (0.59-0.88)	*Age at the first dose:*<br>all ages:<br>aOR = 0.79 (0.74–0.85)<br>11-14:<br>aOR = 0.67 (0.45-1.02)<br>15-18:<br>aOR = 0.80 (0.72-0.89)<br>19-22:<br>aOR = 0.80 (0.72-0.90)<br>23-27:<br>aOR = 0.80 (0.67-0.96)	*Age at the first dose:*<br>all ages:<br>aOR = 0.95 (0.89–1.02)<br>11-14:<br>aOR = 0.84 (0.52-1.36)<br>15-18:<br>aOR = 0.98 (0.87-1.10)<br>19-22:<br>aOR = 0.98 (0.89-1.08)<br>23-27:<br>aOR = 0.86 (0.74-1.02)
	CIN3/AIS	Age in 2007, remoteness and<br>SES	*Age at the first dose:*<br>all ages:<br>aHR = 0.69 (0.58–0.81)<br>≤16:<br>aHR = 0.43 (0.23–0.79)<br>17-19:<br>aHR = 0.56 (0.44–0.73)<br>20-23:<br>aHR = 0.71 (0.54–0.93)	*Age at the first dose:*<br>all ages:<br>aHR = 1.17 (0.92–1.48)<br>≤16:<br>aHR = 0.48 (0.11–2.03)<br>17-19:<br>aHR = 1.11 (0.72–1.70)<br>20-23:<br>aHR = 1.18 (0.82–1.69)	*Age at the first dose:*<br>all ages:<br>aHR = 1.41 (1.12–1.77)<br>≤16:<br>1.20 (0.37–3.92)<br>17-19:<br>aHR = 1.38 (0.89–2.15)<br>20-23:<br>aHR = 1.30 (0.91–1.85)

Brotherton 2015 [11]	CIN2+		*Age at the first dose:*<br>all ages:<br>aHR = 0.71 (0.64–0.80)<br>≤16:<br>aHR = 0.59 (0.41–0.85)<br>17-19:<br>aHR = 0.61 (0.51–0.72)<br>20-23:<br>aHR = 0.75 (0.62–0.91)<br>24-26:<br>aHR = 1.07 (0.82–1.40)	*Age at the first dose:*<br>all ages:<br>aHR = 1.21 (1.02–1.44)<br>≤16:<br>aHR = 0.70 (0.32–1.53)<br>17-19:<br>aHR = 1.01 (0.74–1.37)<br>20-23:<br>aHR = 1.33 (1.03–1.71)<br>24-26:<br>aHR = 1.41 (0.95–2.08)	*Age at the first dose:*<br>all ages:<br>aHR = 1.19 (0.99–1.43)<br>≤16:<br>aHR = 0.83 (0.33–2.05)<br>17-19:<br>aHR = 1.02 (0.72–1.45)<br>20-23:<br>aHR = 1.16 (0.88–1.53)<br>24-26:<br>aHR = 1.53 (1.06–2.21)
	CIN2		*Age at the first dose:*<br>all ages:<br>aHR = 0.75 (0.65–0.86)<br>≤16:<br>aHR = 0.74 (0.48–1.16)<br>17-19:<br>aHR = 0.66 (0.53–0.81)<br>20-23:<br>aHR = 0.80 (0.62–1.04)	*Age at the first dose:*<br>all ages:<br>aHR = 1.22 (0.97–1.54)<br>≤16:<br>aHR = 1.00 (0.42–2.38)<br>17-19:<br>aHR = 0.92 (0.61–1.41)<br>20-23:<br>aHR = 1.45 (1.03–2.02)	*Age at the first dose:*<br>all ages:<br>aHR = 0.98 (0.75–1.29)<br>≤16:<br>aHR = 0.55 (0.13–2.27)<br>17-19:<br>aHR = 0.66 (0.38–1.15)<br>20-23:<br>aHR = 1.03 (0.69–1.54)
	High-grade		*Age at the first dose:*<br>all ages:<br>aHR = 0.53 (0.47–0.60)<br>≤16:<br>aHR = 0.55 (0.35–0.86)<br>17-19:<br>aHR = 0.60 (0.50–0.73)<br>20-23:<br>aHR = 0.49 (0.39–0.63)	*Age at the first dose:*<br>all ages:<br>aHR = 0.63 (0.50–0.80)<br>≤16:<br>aHR = 1.27 (0.62–2.58)<br>17-19:<br>aHR = 0.61 (0.40–0.93)<br>20-23:<br>aHR = 0.65 (0.45–0.93)	*Age at the first dose:*<br>all ages:<br>aHR = 0.44 (0.32–0.59)<br>≤16:<br>aHR = 0.97 (0.35–2.70)<br>17-19:<br>aHR = 0.48 (0.28–0.82)<br>20-23:<br>aHR = 0.45 (0.29–0.70)
	Low-grade		*Age at the first dose:*<br>all ages:<br>aHR = 0.73 (0.68–0.78)<br>≤16:<br>aHR = 0.85 (0.68–1.06)<br>17-19:<br>aHR = 0.85 (0.77–0.94)<br>20-23:<br>aHR = 0.58 (0.50–0.67)	*Age at the first dose:*<br>all ages:<br>aHR = 0.52 (0.44–0.61)<br>≤16:<br>aHR = 0.48 (0.25–0.90)<br>17-19:<br>aHR = 0.70 (0.55–0.89)<br>20-23:<br>aHR = 0.47 (0.36–0.62)	*Age at the first dose:*<br>all ages:<br>aHR = 0.48 (0.40–0.58)<br>≤16:<br>aHR = 0.82 (0.43–1.55)<br>17-19:<br>aHR = 0.61 (0.46–0.82)<br>20-23:<br>aHR = 0.43 (0.32–0.58)

Hofstetter 2016<br>[23]	Abnormal	Age as of January 1, 2007, language, insurance, clinic type; abnormal baseline cervical cytology result, baseline Chlamydia screening	Age at the first dose:<br>all ages:<br>aHR = 0.58 (0.48–0.69)<br>11-14:<br>aHR = 0.12 (0.04-0.35)<br>15-16:<br>aHR = 0.58 (0.40-0.84)<br>17-18:<br>aHR = 0.58 (0.44-0.77)<br>19-20:<br>aHR = 0.69 (0.49-0.96)	*Age at the first dose:*<br>all ages:<br>aHR = 0.81 (0.66–0.99)<br>11-14:<br>aHR = 0.28 (0.07-1.12)<br>15-16:<br>aHR = 0.49 (0.28-0.85)<br>17-18:<br>aHR = 0.94 (0.69-1.29)<br>19-20:<br>aHR = 0.83 (0.59-1.16)	*Age at the first dose:*<br>all ages:<br>aHR = 1.05 (0.88–1.26)<br>11-14:<br>aHR = 0.41 (0.10-1.63)<br>15-16:<br>aHR = 1.45 (0.88-2.37)<br>17-18:<br>aHR = 1.03 (0.77-1.39)<br>19-20:<br>aHR = 1.07 (0.81-1.42)
Kim 2016<br>[24]	High-grade	— —	aOR = 0.48 (0.28–0.81)	aOR = 0.17 (0.02–1.20)	aOR = 0.45 (0.11–1.83)
	Low-grade		aOR = 0.74 (0.64–0.85)	aOR = 1.15 (0.90–1.49)	aOR = 0.90 (0.64–1.24)
	Abnormal		aOR = 0.72 (0.63–0.82)	aOR = 1.08 (0.84–1.38)	aOR = 0.86 (0.62–1.18)
Silverberg 2018<br>[34]	CIN3+	— —	RR = 0.68 (0.52–0.90)	RR = 1.02 (0.71–1.48)	RR = 0.94 (0.68–1.30)
	CIN2+		RR = 0.78 (0.66–0.91)	RR = 1.02 (0.82–1.28)	RR = 0.89 (0.73–1.09)
Dehlendorff 2018<br>[30]	CIN2+	Attained age, mother's education, and country	Age at the first dose:<br>13-16:<br>aIRR = 0.23 (0.11–0.49);<br>17-19:<br>aIRR = 0.65 (0.41–1.03);<br>20-29:<br>aIRR = 1.31 (0.97–1.76)	Age at the first dose:<br>13-16:<br>aIRR = 0.44 (0.10–2.03);<br>17-19:<br>aIRR = 0.65 (0.25–1.74);<br>20-29:<br>aIRR = 1.56 (1.15–2.11)	Age at the first dose:<br>13-16:<br>aIRR = 0.23 (0.01–5.24);<br>17-19:<br>aIRR = 0.58 (0.15–2.19);<br>20-29:<br>aIRR = 1.56 (1.13–2.15)
Brotherton 2019<br>[37]	CIN3/AIS+histopathology	Age, area of residence, socioeconomic status.	aHR = 0.43 (0.35–0.53)	aHR = 0.42 (0.27–0.64)	aHR = 0.66 (0.41–1.06)
	CIN2+/AIS		aHR = 0.59 (0.54–0.65)	aHR = 0.61 (0.52–0.72)	aHR = 0.65 (0.52–0.81)

Verdoodt 2020 [46]	CIN3+	Maternal education, attained age (time-scale)	*Age at the first dose:* *all ages:* aIRR = 0.37 (0.30–0.45) <23: aIRR = 0.33 (0.26-0.42) ≥23: aIRR = 0.51 (0.34-0.77)	*Age at the first dose:* *all ages:* aIRR = 0.38 (0.22–0.66) <23: aIRR = 0.37 (0.21-0.66) ≥23: aIRR = 0.44 (0.12-1.60)	*Age at the first dose:* *all ages:* aIRR = 0.38 (0.14–0.98) <23: aIRR = 0.32 (0.11-0.99) ≥23: aIRR = 0.61 (0.10-3.76)
	CIN2+		*Age at the first dose:* *all ages:* aHR = 0.43 (0.36–0.51) <23: aHR = 0.40 (0.33-0.49) ≥23: aHR = 0.57 (0.39-0.83)	*Age at the first dose:* *all ages:* aHR = 0.49 (0.32–0.76) <23: aHR = 0.45 (0.28-0.73) ≥23: aHR = 0.71 (0.26-1.92)	*Age at the first dose:* *all ages:* aHR = 0.34 (0.13–0.87) <23: aHR = 0.35 (0.13-0.92) ≥23: aHR = 0.26 (0.02-3.92)
Johnson 2020 [42]	CIN2+	Site, race/ethnicity, insurance status, age at vaccination	aOR = 0.26 (0.20–0.35)	aOR = 0.45 (0.30–0.69)	aOR = 0.53 (0.37–0.76)
Rodriguez 2020 [45]	CIN2/3	Region, history of sexually transmitted diseases, pregnancy.	*Age at the first dose:* <15: aHR = 0.71 (0.37-1.38); 15-19: aHR = 0.66 (0.55–0.81); ≥20: aHR = 0.96 (0.77-1.20)	*Age at the first dose:* <15: aHR = 0.46 (0.13-1.62); 15-19: aHR = 0.72 (0.54–0.95); ≥20: aHR = 1.02 (0.75-1.38)	*Age at the first dose:* <15: aHR = 0.87 (0.28-2.68); 15-19: aHR = 0.64 (0.47–0.88); ≥20: aHR = 1.16 (0.89-1.52)
	High-grade		*Age at the first dose:* <15: aHR = 0.99 (0.62-1.58); 15-19: aHR = 0.84 (0.73-0.97); ≥20: aHR = 1.08 (0.90-1.30)	*Age at the first dose:* <15: aHR = 0.94 (0.46-1.95); 15-19: aHR = 0.87 (0.70-1.07); ≥20: aHR = 0.92 (0.70-1.20)	*Age at the first dose:* <15: aHR =1.09 (0.48-2.45); 15-19: aHR = 0.87 (0.69-1.08); ≥20: aHR = 1.07 (0.84-1.35)
**Genital warts in women and men**					

Herweijer 2014 [16]	Genital warts	Age, parental education level	10-16:<br>aIRR = 0.18 (0.15–0.22)<br>17-19:<br>aIRR = 0.23 (0.18–0.29)<br>10-19:<br>aIRR = 0.20 (0.17-0.23)	10-16:<br>aIRR = 0.29 (0.21-0.40)<br>17-19:<br>aIRR = 0.35 (0.26-0.47)<br>10-19:<br>aIRR = 0.32 (0.26-0.40)	10-16:<br>aIRR = 0.31 (0.20-0.49)<br>17-19:<br>aIRR = 0.71 (0.55-0.92)<br>10-19:<br>aIRR = 0.54 (0.43-0.68)
Blomberg 2015 [19]	Genital warts	— —	crude incidence rate<br>Per 100000 person-years:<br>Case: 67.2 (62.0-74.2)<br>Control: 655.6 (645.0-666.4)	crude incidence rate<br>Per 100000 person-years:<br>Case: 146.4 (129.6-165.2)<br>Control: 655.6 (645.0-666.4)	crude incidence rate<br>Per 100000 person-years:<br>Case: 333.3 (301.0-369.1)<br>Control: 655.6 (645.0-666.4)
Dominiak-Felden 2015 [20]	Genital warts	Age	aIRR = 0.12 (0.07–0.21)	aIRR = 0.34 (0.14–0.83)	aIRR = 0.63 (0.35–1.16)
Perkins 2017 [29]	Genital warts	— —	crude incidence rate<br>Per 100000 person-years:<br>Case: 1500<br>Control: 2170	crude incidence rate<br>Per 100000 person-years:<br>Case: 1760<br>Control: 2170	crude incidence rate<br>Per 100000 person-years:<br>Case: 1900<br>Control: 2170
Navarro-Illana 2017 [28]	Genital warts	Age, calendar year, health department	aRR = 0.24 (0.15–0.34)	aRR = 0.36 (0.14–0.68)	aRR = 0.39 (0.13–0.80)
Hariri 2018 [31]	Genital warts	Race/ethnicity, health plan (site), age at beginning of health plan enrollment, age at beginning of study period, age at first evidence of probable sexual activity, Age at the first dose of HPV vaccine, indicator for whether the person was continuously enrolled from index date to the end of the study period, months enrolled in the health plan, indicator for whether the person had any preventive health visits, Medicaid enrollment, oral contraceptive use, and	*6 month buffer from last dose:*<br>aHR = 0.23 (0.17–0.31)<br>*12 month buffer from first dose:*<br>aHR = 0.20 (0.15–0.27)	*6 month buffer from last dose:*<br><6 month interval:<br>aHR = 0.91 (0.59-1.41)<br>≥6 month interval:<br>aHR = 0.32 (0.17–0.59)<br>*12 month buffer from first dose:*<br><6 month interval:<br>aHR = 0.72 (0.41, 1.25)<br>≥6 month interval:<br>aHR = 0.24 (0.13, 0.44)	*6 month buffer from last dose:*<br>aHR = 0.81 (0.60–1.08)<br>*12 month buffer from first dose:*<br>aHR = 0.32 (0.20–0.52)

		history of pregnancy test, chlamydia test, and gonor rhea test			
Zeybek 2018 [36]	Genital warts	Sex, region, the history of sexually transmitted diseases	*Age at the first dose:*<br><15:<br>aRR = 0.78 (0.46–1.35)<br>15–19:<br>aRR = 0.58 (0.49–0.70)<br>≥20:<br>aRR = 1.11 (0.91–1.35)	*Age at the first dose:*<br><15:<br>aRR = 1.36 (0.65–2.86)<br>15–19:<br>aRR = 0.67 (0.51–0.89)<br>≥20:<br>aRR = 1.15 (0.87–1.51)	*Age at the first dose:*<br><15:<br>aRR = 0.80 (0.34–1.90)<br>15–19:<br>aRR = 0.65 (0.49–0.85)<br>≥20:<br>aRR = 0.96 (0.72–1.28)
Willows 2018 [35]	Genital warts	Birth date, neighborhood of residence, previous hospitalization, previous physician visit	*Age at the first dose:*<br>9-18:<br>aHR = 0.4 (0.3–0.7)<br>≥19 and not sexually active:<br>aHR = 3.1 (0.6-14.8)<br>≥ 19 and sexually active:<br>aHR = 2.5 (1.7–3.6)	*Age at the first dose:*<br>9-18:<br>aHR = 1.4 (0.6–3.3)<br>≥19 and not sexually active:<br>— —<br>≥19 and sexually active:<br>aHR = 3.0 (1.6–5.7)	*Age at the first dose:*<br>9-18:<br>aHR = 0.6 (0.2–1.8)<br>≥19 and not sexually active:<br>aHR = 3.1 (0.2-44.7)<br>≥19 and sexually active:<br>aHR = 3.7 (2.1–6.8)
Baandrup 2021 [48]	Genital warts	Attained age, socioeconomic status, calendar time.	*Age at the first dose:*<br>12-14:<br>aIRR = 0.16 (0.15-0.18)<br>15-16:<br>aIRR = 0.20 (0.18-0.22)<br>17-18:<br>aIRR = 0.29 (0.25-0.33)<br>≥19:<br>aIRR = 0.76 (0.71-0.81)	*Age at the first dose:*<br>12-14:<br>aIRR = 0.22 (0.18-0.26)<br>15-16:<br>aIRR = 0.32 (0.26-0.38)<br>17-18:<br>aIRR = 0.49 (0.39-0.62)<br>≥19:<br>aIRR = 1.03 (0.95-1.12)	*Age at the first dose:*<br>12-14:<br>aIRR = 0.29 (0.22-0.38)<br>15-16:<br>aIRR = 0.38 (0.29-0.49)<br>17-18:<br>aIRR = 0.56 (0.42-0.73)<br>≥19:<br>aIRR = 1.36 (1.24-1.49)
Reyburn 2023 [53]	Genital warts	Age, ethnicity and smoking	aPR = 1.28 (0.37, 4.48)	aPR = 0.61 (0.08, 4.95)	aPR = 0.37 (0.05, 2.95)

Abbreviations: HPV, human papillomavirus; aRR, adjusted risk ratios; aHR, adjusted hazard ratios; aIRR, adjusted incidence rate ratios; aPR, adjusted prevalence ratios; aOR, adjusted odds ratios; CIN, cervical intraepithelial neoplasia; AIS, adenocarcinoma in situ.

Supplementary Table S4. Clinical effectiveness of Nonavalent HPV vaccine by number of doses

Endpoint/ Vaccine/	Outcome	Adjustment	HR (95%CI), p value

Study			3 doses/control	2 doses/control	1 dose/control
		**Cervical HPV infection in women**			
Barnabas 2022 [50]	Persistent HPV 16/18 infection for ≥4 months in 18-month follow-up	— —	— —	— —	HR = 0.025 (0.003-0.183)
	Persistent HPV 16/18/31/33/45/52/58 for ≥4 months in 18-month follow-up		— —	— —	HR = 0.111 (0.039-0.315)
Barnabas 2023 [51]	Persistent HPV 16/18 infection for ≥4 months in 3-year follow-up	— —	— —	— —	HR = 0.012 (0.002-0.087)
	Persistent HPV 16/18/31/33/45/52/58 for ≥4 months in 3-year follow-up		— —	— —	HR = 0.045 (0.018-0.110)
	Persistent HPV 31/33/45 infection for ≥4 months in 3-year follow-up		— —	— —	HR = 0.085 (0.031-0.237)

Abbreviations: HPV, human papillomavirus; HR, hazard ratios.
